# Role of the histone variant H2A.Z.1 in memory, transcription, and alternative splicing is mediated by lysine modification

**DOI:** 10.1038/s41386-024-01817-2

**Published:** 2024-02-16

**Authors:** Anas Reda, Luca A. Hategan, Timothy A. B. McLean, Samantha D. Creighton, Jian Qi Luo, Sean En Si Chen, Shan Hua, Stephen Winston, Isaiah Reeves, Aditya Padmanabhan, Tarkan A. Dahi, Firyal Ramzan, Mark A. Brimble, Patrick J. Murphy, Brandon J. Walters, Gilda Stefanelli, Iva B. Zovkic

**Affiliations:** 1 Department of Cell & Systems Biology, University of Toronto, Toronto, ON M5S 3G3 Canada; 2 Department of Psychology, University of Toronto Mississauga, Mississauga, ON L5L 1C6 Canada; 3 Departments of Biology and Biomedical Genetics, University of Rochester Medical Center, Rochester, NY 14642 USA; 4 Department of Surgery and Graduate school of Biomedical Sciences, St. Jude Children’s Research Hospital, Memphis, TN 38105 USA; 5 Department of Biology, University of Toronto Mississauga, Mississauga, ON L5L 1C6 Canada; 6 Department of Biology, University of Waterloo, Waterloo, ON N2L 3G1 Canada; 7 Department of Host-Microbe Interactions, St. Jude Children’s Research Hospital, Memphis, TN 38105 USA; 8 Department of Biology, University of Ottawa, Ottawa, ON K1N 6N5 Canada

**Keywords:** Epigenetics and behaviour, Long-term memory

## Abstract

Creating long-lasting memories requires learning-induced changes in gene expression, which are impacted by epigenetic modifications of DNA and associated histone proteins. Post-translational modifications (PTMs) of histones are key regulators of transcription, with different PTMs producing unique effects on gene activity and behavior. Although recent studies implicate histone variants as novel regulators of memory, effects of PTMs on the function of histone variants are rarely considered. We previously showed that the histone variant H2A.Z suppresses memory, but it is unclear if this role is impacted by H2A.Z acetylation, a PTM that is typically associated with positive effects on transcription and memory. To answer this question, we used a mutation approach to manipulate acetylation on H2A.Z without impacting acetylation of other histone types. Specifically, we used adeno-associated virus (AAV) constructs to overexpress mutated H2A.Z.1 isoforms that either mimic acetylation (acetyl-mimic) by replacing lysines 4, 7 and 11 with glutamine (KQ), or H2A.Z.1 with impaired acetylation (acetyl-defective) by replacing the same lysines with alanine (KA). Expressing the H2A.Z.1 acetyl-mimic (H2A.Z.1^KQ^) improved memory under weak learning conditions, whereas expressing the acetyl-defective H2A.Z.1^KA^ generally impaired memory, indicating that the effect of H2A.Z.1 on memory depends on its acetylation status. RNA sequencing showed that H2A.Z.1^KQ^ and H2A.Z.1^KA^ uniquely impact the expression of different classes of genes in both females and males. Specifically, H2A.Z.1^KA^ preferentially impacts genes involved in synaptic function, suggesting that acetyl-defective H2A.Z.1 impairs memory by altering synaptic regulation. Finally, we describe, for the first time, that H2A.Z is also involved in alternative splicing of neuronal genes, whereby H2A.Z depletion, as well as expression of H2A.Z.1 lysine mutants influence transcription and splicing of different gene targets, suggesting that H2A.Z.1 can impact behavior through effects on both splicing and gene expression. This is the first study to demonstrate that direct manipulation of H2A.Z post-translational modifications regulates memory, whereby acetylation adds another regulatory layer by which histone variants can fine tune higher brain functions through effects on gene expression and splicing.

## Introduction

Formation of long-term memories is dependent on learning-induced changes in transcription and associated modifications of histone proteins [1]. Histones regulate accessibility of genes for transcription by packaging ~147 base pair segments of DNA around 2 copies of histones H2A, H2B, H3 and H4 to form nucleosomes, the building blocks of chromatin. Histones modify gene expression through post-translational modifications (PTMs) of their N-terminal tails, or differential inclusion of histone variants into nucleosomes [2]. Histone variants are functionally diverse counterparts of canonical histones that alter nucleosome function to modify gene expression and alternative splicing [3], and recent evidence shows that variants of the H2A histone family regulate memory and neuronal transcription [4–13]. Histone variants are especially important for neural function because expression of canonical histones is coupled to cell division, which reduces their abundance adulthood [14, 15], whereas replication-independent histone variants become the primary source of histones in the adult brain [15]. Despite their critical role in neuronal chromatin, untangling the function of histone variants is complex because they are encoded by multiple genes and are typically studied without considering PTMs that modify their function.

Histone H2A.Z is a highly conserved H2A variant that suppresses memory in male mice [2, 8, 9, 11–13]. H2A.Z is encoded by 2 genes that produce histones H2A.Z.1 (encoded by *H2afz*) and H2A.Z.2 (encoded by *H2afv*) that differ in only 3 amino acids [16]. Most studies focus on H2A.Z.1, the primary form of H2A.Z in cultured rat neurons [17], whose depletion improves memory [2, 8, 9, 11–13]. Although H2A.Z.2 function in memory has not been directly studied, inducible neuronal deletion of both H2A.Z-encoding genes improved aversive and non-aversive memory in male mice [8, 9] and improved only non-aversive memory in females, indicating that effects of H2A.Z on memory are sex and task specific [9].

Levels of acetylated H2A.Z increase 24 h and 30 days after fear conditioning [6], suggesting that H2A.Z acetylation (AcH2A.Z) may be beneficial for memory. Although the functional role of H2A.Z acetylation in memory has not been directly studied, acetylation generally improves memory [18], suggesting that acetylation of H2A.Z may also promote memory formation. However, tools for manipulating histone acetylation primarily utilize histone deacetylase (HDAC) enzymes, which alter acetylation across various histones and non-histone proteins, making it difficult to assign outcomes to any particular histone. Indeed, trichostatin A (TSA) is a widely used HDAC inhibitor that enhances memory in wild-type mice and improves pathology and memory in mouse models of Alzheimer’s disease [18–20]. Its effects have been attributed largely to changes in H3 and H4 acetylation, but we found that TSA also causes a dramatic increase in histone H2A.Z acetylation [6], suggesting that some benefits of HDAC inhibitors may involve H2A.Z acetylation.

The goal of the current study is to assess the functional role of H2A.Z.1 acetylation in memory independently from acetylation of other histones. H2A.Z is most commonly acetylated at lysines 9, 7 and 11 (K9, K7 and K11) [16]. To investigate the functional relevance of these lysines for memory, we applied a mutation strategy to replace these lysines with glutamine (KQ) to mimic acetylation, or with alanine (KA) to prevent acetylation. Glutamine is structurally similar to acetylated lysine and can mimic functional effects of histone acetylation in several model systems, whereas alanine cannot be acetylated and thus acts as an acetyl-defective mutant [21, 22]. We focused specifically on H2A.Z.1 because this is the primary form of H2A.Z in rat cortical neurons [17] and because H2A.Z.1 depletion enhances memory [9, 11–13].

## Methods

### Animals

Male and female conditional-inducible H2A.Z knockout (H2A.Z^KO^) mice floxed for both H2A.Z.1 (*H2afz*) and H2A.Z.2 (*H2afv*) were purchased from Riken and maintained in our colony (RBRC #05765) at the University of Toronto Mississauga. Mice were housed in groups on a 12 h light cycle (lights on at 8 am) with *ad libitum* access to food and water. All procedures were approved by the University of Toronto Animal Care Committee and complied with institutional guidelines and the Canadian Council on Animal Care.

### AAV production and constructs

H2A.Z deletion in the hippocampus was achieved using an AAV ITR-flanked vector encoding Cre recombinase open reading frame (ORF) driven by the Synapsin (SYN) promoter (Syn-Cre). *H2afz* was overexpressed with a vector encoding H2A.Z.1 ORF fused to 3x-Myc tag and lacking the stop codon under CMV promoter, followed by p2A auto-cleaving sequence and GFP ORF. The 3x-Myc sequence followed by p2A GFP was used as control. To obtain acetyl-defective (KA) and acetyl-mimic (KQ) mutants, Lysine 4, 7 and 11 were mutated into Alanine (GCC) or Glutamine (CAA; Supplementary Fig. 2A, B).

### Stereotaxic surgery for viral delivery

Mice were anesthetized with isoflurane and secured in a Kopf stereotaxic apparatus. Viral particles were bilaterally delivered into the hippocampus (anterior/posterior −2; medial/lateral 1.5; dorsal/ventral −1.6) at a rate of 225 nl/min. Syn-Cre viral particles were mixed with 3-Myc-H2A.Z.1 WT, KA or KQ or 3-Myc only as control and a total volume of 2 ul of virus mix was injected in each hemisphere. Titers were matched to the least concentrated viral vector (6.60 e12 viral genomes (VG)/ml). For the control condition (3x-Myc only), virus was mixed with PBS to allow for titer and volume matching among conditions so that all mice received the same volume of viral injections. Mice were allowed 2 weeks to recover before testing (Supplementary Fig. 2C).

### Object location memory (OLM)

Mice were transported to the testing room and given 30 min to acclimate. Before testing, all mice were handled and habituated to an empty testing apparatus (open-field; 45 × 45 × 30 cm) for 10 min on two consecutive days. Spatial and contextual cues were readily available in the testing room. Mice underwent one 5-min (suboptimal) or 10-min (standard) training phase, in which they were presented with 2 objects in 2 corners of the open-field, away from the walls. Memory was tested after 24 h with a 5-min exposure to the same 2 objects, with one presented in a new location. Memory was inferred from the preferential exploration of the object in the novel compared to the familiar side, quantified as the discrimination ratio [DR = (seconds spent in novel location exploration – familiar location exploration)/(total seconds in object exploration)]. Mice were first trained with a standard 10 min protocol. One week later, a subset of mice were trained on the suboptimal 5 min protocol using different objects.

### Fear conditioning

One week after the last OLM task, mice underwent 2 contextual fear conditioning sessions administered 1 week apart. Mice were transported to the testing room and placed into test chambers (9.8 in boxes; designed for mice) equipped with an electrified grid floor (Coulbourn Instruments, Holliston, MA, USA). During training, mice were given 2 min to explore, followed by one or two 0.3 mA (2 s) foot-shocks an additional minute of exploration. One week later, mice were re-trained with a single 0.5 mA instead of a 0.3 mA shock. Memory was tested in the same context 24 h after each training and freezing (scored by FreezeFrame, Coulbourn Instruments) was used as an index of memory.

### Primary hippocampal neuronal culture

Hippocampal neurons were prepared from E17 mice floxed for both H2A.Z.1 (*H2afz*) and H2A.Z.2 (*H2afv*), as previously described [8]. Hippocampi were washed with Hank’s Balanced Salt Solution (1x HBSS Gibco#14175-095) and trypsinated for 30 s (0.25% Trypsin, Life Technologies#15050065), then washed with HBSS. Trypsin was inhibited with dissecting medium (10% normal horse serum, Life Technologies#26050070) in DMEM (Life Technologies#11995065). Neurons were dissociated by triturating and diluted in Neurobasal Medium (L-Glutamine) (Gibco#21103-049) with 20 mM L-glutamine (Gibco 25030-081), 1x Penicillin-Streptomycin (Sigma P4333-100ML) and 1xB27 (Gibco 17504-044) and plated in 6-well plates. Neurons were infected with Syn-Cre on 1 day in vitro (DIV) and harvested on DIV8.

### Chromatin immunoprecipitation (ChIP)

#### ChIP-qPCR

Whole hippocampus from the left hemisphere was crosslinked in 500 μL of 1% formaldehyde in PBS with protease inhibitor cocktail (Cell Signaling#5872) for 5 min at RT, then quenched with 1.25 M glycine for 5 min at RT. Tissue was washed 6x with ice-cold PBS and homogenized in 300 μL ChIP lysis buffer (50 mM Tris pH 8.0, 1% SDS, 10 mM EDTA) with protease inhibitor cocktail. Tissue homogenates were sonicated for 10 s at 40% power and sheared using a Bioruptor (40 cycles, 30 s on 30 s off; Diagenode), then centrifuged at 17,000 × *g* for 5 min at 4 °C to clear insoluble material. Supernatant was aliquoted, diluted 1:10 with ChIP dilution buffer (16 mM Tris pH 8.0, 0.01% SDS, 1% Triton X-100, 1.2 mM EDTA, 170 mM NaCl), and incubated with 20uL of pre-blocked Protein G beads (Millipore #16-662) and 1uL of anti-H2A.Z (Millipore #ABE1348) or anti-acH2A.Z (Millipore #ABE1363) overnight at 4 °C on a rotating platform. Immunoprecipitates were washed sequentially with low-salt (0.1% SDS, 1% Triton X-100, 2 mM EDTA, 20 mM Tris-HCl pH 8.0, 150 mM NaCl), high-salt (0.1% SDS, 1% Triton X-100, 2 mM EDTA, 20 mM Tris-HCl pH 8.0, 500 mM NaCl), LiCl (0.25 M LiCl, 1% IGEPAL CA630, 1% sodium deoxycholate, 1 mM EDTA, 10 mM Tris pH 8.0), and TE (10 mM Tris-HCl pH 8.0, 1 mM EDTA) for 5 min at RT. Immunoprecipitates and inputs were de-crosslinked for 2 h at 65°C in TE (10 mM Tris-HCl, 1 mM EDTA) with 1% SDS and 9.25ug proteinase K (Roche #03115887001), then incubated at 95°C for 10 minutes. Samples were cooled to RT and DNA was purified with a PCR purification kit (Bio Basic #BS664). ChIP data were calculated as %input. Acetylated H2A.Z was normalized to total H2A.Z by dividing %input (acH2A.Z) by %input (H2A.Z).

#### RNA-seq

RNA was extracted using the RNeasy Mini Kit (Qiagen). Samples were sequenced at CD Genomics using Illumina HiSeq4000 at a PE read depth of ~30 million. Raw PE reads were quality controlled using FastQC (v0.11.9). Accession number for raw sequencing data is GEO: GSE242179.

#### DEG analysis

Reads were aligned to the *Mus musculus* GRCm39 reference genome (Ensembl). Reference genome was indexed in R using the Rsubread library and reads were aligned using Rsubread align in R (version 2.12.3, R version 4.2.3) with paired-end settings. Adapters were trimmed using soft-clipping in Rsubread [23]. Aligned reads were counted using featureCounts in the Rsubread library (version 2.12.3). Quality control on the counts was performed using RNASeqQC in R (version 0.1.4). Genes with count sum below 20 across all samples were removed prior to analysis. We performed PCA on the samples, as well as Mahalanobis distance metrics to assess for potential outlier samples. Differential expression analysis was performed in DESeq2 in R (version 1.38.3) using adjp<0.05 and volcano plots were generated using EnhancedVolcano library in R (version 1.16.0). Overlap data for Venn diagrams were generated using webtool from Bioinformatics and Evolutionary Genomics (https://bioinformatics.psb.ugent.be/webtools/Venn/).

#### Quantile plots

DEG tables from Myc controls vs. H2A.Z^KO^ mice were sorted by Log_2_FC value in increasing order. Quintiles were generated by grouping genes from H2A.Z^KO^, H2A.Z.1^WT^, H2A.Z.1^KA^, and H2A.Z.1^KQ^ groups based on Log_2_FC-ordered DEGs from the Myc vs. H2A.Z^KO^ group. Box-whisker plots were generated with those Log_2_FC values using ggplot2 in R. Difference between the mean Log_2_FC between all four groups within each quantile were assessed with a 1-way ANOVA followed by Tukey’s HSD post-hoc with multiple comparison correction.

#### Heat maps

DEGs from the H2A.Z.1^KA^ and H2A.Z.1^KQ^ groups were filtered based on a padj<0.05 and their union set was taken. A differential between normalized gene counts from H2A.Z.1^KA^ and H2A.Z.1^KQ^ was calculated. Genes from the union set were matched to the row name of the normalized count matrix from DESeq2. The mean expression of each gene across each replicate was calculated for H2A.Z.1^KA^ and H2A.Z.1^KQ^, respectively. The difference between these values were calculated and the genes were sorted by the differential in descending order. Top 100 genes upregulated in H2A.Z.1^KA^ and downregulated in H2A.Z.1^KQ^, and top 100 genes upregulated in H2A.Z.1^KQ^ and downregulated in H2A.Z.1^KA^ (and same genes in H2A.Z.1^WT^) were then Z-scaled and plotted using ComplexHeatmap in R (v. 2.17.0) [24]. Unsupervised clustering was conducted within ComplexHeatmap and dendrograms grouping samples and replicates (columns), and genes (rows) were plotted.

#### RNA-seq GO analysis

Gene ontology pathway analyses were conducted using ShinyGo, which compares random distribution of chromosomes to all background genes using ChiSquared. Significant differences between user inputted genes and all other background genes in the genome are analyzed using *t*-tests corrected for multiple comparisons [25].

#### DTU analysis

Quality controlled fastq files were trimmed using trimmomatic (version 0.49) against TruSeq3-PE.fa (trimmomatic, max 4 mismatches, minimum length=50 bp). Trimmed fastq files were again quality controlled using FastQC (v0.11.9) and fastq files were mapped to the indexed GRCm39 using Salmon (version 1.9.0) with default settings. Transcript usage and alternative splicing was analyzed using the IsoformSwitchAnalyzeR library (version 2.1.3). Briefly, quantification files were imported using the standard import pipeline [26]. Isoform usage was tested on retained samples with DEXSeq implemented in IsoformSwitchAnalyzeR [27–29]. Alternative splicing analysis was conducted with spliceR implemented in IsoformSwitchAnalyzeR, and the type of alternative splicing event occurring was visualized using whisker plots [30]. Volcano plots relating differential isoform fraction (dIF) to the log_10_(pAdj) values were generated using ggplot2 in R (version 3.4.1) from the output of DEXSeq in IsoformSwitchAnalyzeR. To generate plots relating differential gene expression with isoform switches, log_2_ fold changes from DESeq2 called genes were plotted against the dIF of transcripts called from DEXSeq using ggplot2. Only genes with more than one documented splice isoform and showing expression in our data set were included in this comparison.

#### Statistics

Behavioral data were analyzed with a two-way ANOVA using SPSS (IBM), with Sex and Virus (Myc, H2A.Z^KO^, H2A.Z^WT^, H2A.Z^KA^, H2A.Z^KQ^) as independent variables. Significance was set to p ≤ 0.05. Post hoc analyses were conducted using Two-Stage Linear Step-Up Procedure of Benjamini, Krieger and Yekutieli to control for False Discovery Rate (FDR), with q value set to 0.05.

## Results

### H2A.Z acetylation associates with accessible regions

**Fig. 1:**
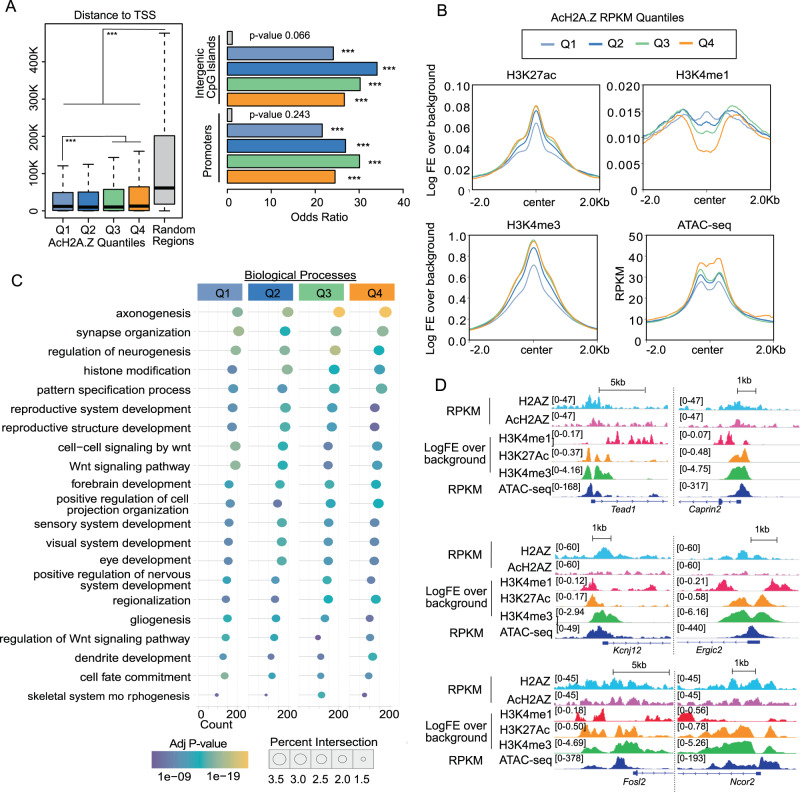
Acetylated H2A.Z occurs over accessible regions in hippocampal neurons. **A** Left: Measurements of peak distance to nearest TSS across each H2A.Z enrichment quartile (Peaks for H2A.Z were separated into quartiles according to acetylated H2A.Z levels). All AcH2A.Z peaks are relatively close to TSS, as compared with a random sampling of the genome, and regions within Q3 or Q4 tend to be further from genes than Q1 or Q2. Right: Measurements of AcH2A.Z peaks intersection with promoters or intergenic CpG Islands, a proxy for putative enhancers. Fisher odds ratios are displayed as a bar chart with p-value indicated. P values generated from two tailed hypergeometry testing. ***indicates p < 0.0001. **B** Enrichment for chromatin modifications across quartiles defined in panel A. Regions within Q3 or Q4 tend to have higher levels of H3K4me3, H3K27ac, and chromatin accessibility, as compared with Q1 and Q2, but lower levels of H3K4me1. **C** Gene ontology analysis indicates a significant enrichment for gene pathways associated with neuron and brain function across all quartiles, with the most significant enrichment occurring at the most highly enriched AcH2A.Z loci, those within Q3 and Q4. **D** Genome browser snapshot depicts example genes where AcH2A.Z levels are enriched. AcH2A.Z ChIP seq N = 3.

First, we assessed the chromatin distribution of acetylated H2A.Z (AcH2A.Z) relative to total H2A.Z using ChIP-seq in primary hippocampal neurons. H2A.Z was sorted into quartiles based on AcH2A.Z signal, with Q1 representing the lowest and Q4 denoting the highest level of acetylation (Fig. 1A, left; Supplementary Fig. 1). AcH2A.Z within Q3 or Q4 localized further from genes than H2A.Z with low levels of acetylation (Q1 or Q2). Moreover, H2A.Z at active promoters (indexed by H3K4me3) tends to be acetylated, such that the least acetylated H2A.Z (Q1) is less abundant than H2A.Z in the remaining quartiles (Q2-Q4) (Fig. 1B, D). In contrast, acetylated H2A.Z (Q4) is excluded from enhancers marked by H3K4me1, a modification that does not distinguish between active and inactive states, but is found on active enhancers marked by H3K27ac [31]. To confirm AcH2A.Z occupancy at enhancers and promoters, we showed that AcH2A.Z in each quantile intersects with promoters and intergenic CpG islands, which are used as a proxy for putative enhancers (Fig. 3A, right) [32]. Thus, AcH2A.Z occurs both at promoters and putative enhancers, and the highest AcH2A.Z levels tend to be located further from gene TSS than regions possessing lower levels of AcH2A.Z. Additionally, AcH2A.Z occupied open chromatin regions indexed by ATAC seq (Fig. 1B, D), suggesting that H2A.Z acetylation is associated with permissive transcriptional states. Gene ontology analysis of AcH2A.Z occupancy identified neuron and brain function across all quartiles, with greatest enrichment at the most highly acetylated loci (Fig. 1C).

### Validation of construct incorporation

To assess the functional relevance of H2A.Z acetylation, we used H2A.Z.1 and H2A.Z.2 floxed mice to eliminate endogenous H2A.Z and overexpressed Myc-tagged isoforms of either wild-type (WT), KA, or KQ H2A.Z.1 mutations (Supplementary Fig. 2A–C). First, we confirmed similarities in viral spread of AAVs expressing Cre and each H2A.Z.1 replacement construct in the hippocampus (Supplementary Fig. 2D). Next, we confirmed that Cre injection in floxed mice reduced H2A.Z.1 (*H2afz*) expression when H2A.Z.1 was not replaced (t_16_ = 4.14, p < 0.001; Supplementary Fig. 2E) and reduced H2A.Z.2 (*H2afv*) expression irrespective of H2A.Z.1 replacement (F_3,32_ = 9.95, p < 0.001; all p < 0.05) (Supplementary Fig. 2F). The small degree of H2A.Z deletion likely reflects low sensitivity for detecting neuron-specific knockout in bulk tissue. Indeed, expressing Cre in primary hippocampal neurons from floxed mice robustly reduced H2A.Z levels (Supplementary Fig. 2H), indicating high efficacy of this construct. Finally, we confirmed that H2A.Z.1 expression was higher (F_3,27_ = 11.60, p < 0.001) in mice that received H2A.Z.1^WT^ (standard form of H2A.Z.1), H2A.Z.1^KA^ (acetyl-defective mutant), and H2A.Z.1^KQ^ (acetyl-mimic mutant) expression constructs compared to Myc-tag controls (all p < 0.05), indicating successful construct expression in the brain (Supplementary Fig. 2G).

### Effects of H2A.Z acetyl mutants on memory recall

#### Object location memory (OLM)

**Fig. 2:**
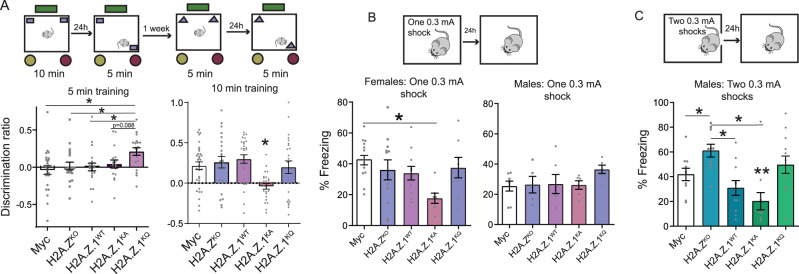
H2A.Z.1 acetyl-mimic promotes memory and acetyl-defective H2A.Z.1 impairs memory. **A** Male and female mice were trained on the object location task for either 10 min (standard training protocol) or 5 min (subthreshold training protocol) and memory was tested 24 h after each training session. On the 5 min task, mice expressing the acetyl-mimic H2A.Z.1^KQ^ had improved memory compared to all other groups (left). On the 10 min task, mice expressing acetyl-defective H2A.Z.1^KA^ had impaired memory compared to all other groups. Sex was not a significant factor, so data are combined for males and females. N/group for females: Myc:13, H2A.Z^KO^:12, H2A.Z.1^WT^:12, H2A.Z.1^KA^:8; H2A.Z.1^KQ^:8. N/group for males 5 min training: Myc:12; H2A.Z^KO^:8, H2A.Z.1^WT^:10, H2A.Z.1^KA^: 10; H2A.Z.1^KQ^:11. N/group for males 10 min training: Myc:19; H2A.Z^KO^:13, H2A.Z.1^WT^:16, H2A.Z.1^KA^:17; H2A.Z.1^KQ^:16. **B** Mice were trained on contextual fear conditioning using a single 0.3 mA foot shock. Female mice expressing acetyl-defective H2A.Z.1^KA^ had impaired memory compared to all other groups. Male mice had lower freezing overall, but freezing did not differ between treatment groups. N/group for females: Myc:13, H2A.Z^KO^:12, H2A.Z.1^WT^:11, H2A.Z.1^KA^:8; H2A.Z.1^KQ^:8. N/group for males: Myc:7, H2A.Z^KO^:5, H2A.Z.1^WT^:6, H2A.Z.1^KA^:6; H2A.Z.1^KQ^:6. **C** A subgroup of male mice were trained with a stronger protocol (two 0.3 mA shocks instead of one 0.3 mA shock). Under these conditions, H2A.Z deletion improved memory, whereas expression of acetyl-defective H2A.Z.1^KA^ impaired memory. * signifies p < 0.05 for comparisons identified by lines between bars; **signifies p < 0.05 for H2A.Z.1^KA^ vs. all other groups N/group = Myc:11; H2A.Z^KO^:10, H2A.Z.1^WT^:11, H2A.Z.1^KA^:10; H2A.Z.1^KQ^:12. Data are expressed as Mean ± SEM. *q < 0.05.

Long-term memory for object location was tested with a standard 10 min training session that produces memory 24 h later, and a subthreshold 5 min training session that fails to produce long-term memory [31] (Fig. 1A). There was no sex difference on the subthreshold task, but mice expressing the acetyl-mimic H2A.Z.1^KQ^ mutant had enhanced learning compared to all other groups (F_4,94_ = 2.885, p = 0.027, all q < 0.05, except H2A.Z.1^KQ^ *vs*. H2A.Z.1^KA^, for which q = 0.088), suggesting that constitutively acetylated H2A.Z.1 is beneficial for consolidating weak memories (Fig. 2A).

Standard training conditions abolished the beneficial effect of H2A.Z.1^KQ^, suggesting that acetylation does not provide additional benefit under training conditions that support learning in control mice. However, the acetyl-defective H2A.Z.1^KA^ mutant impaired memory compared to all other groups (Main effect of Virus: F_4,121_ = 4.57, p = 0.002; all p < 0.05), suggesting that the presence of H2A.Z.1 that cannot be modified is detrimental for memory. In addition, memory was lower in males than in females (Main effect of sex: F_1,121_ = 4.02, p = 0.047; Supplementary Fig. 3), but sex did not interact with H2A.Z lysine mutations. Together, these data show that H2A.Z.1 acetylation is beneficial for memory under weak learning conditions, whereas unacetylated H2A.Z.1 is detrimental for memory under standard learning conditions (Fig. 2A).

#### Contextual fear memory (CFC)

In contrast to object location memory, which is similarly affected by H2A.Z depletion in both sexes [9], we previously showed that effects of H2A.Z deletion on fear memory are sex-specific [9], leading us to assess males and females separately. Mice were fear conditioned using a weak training protocol consisting of a single 0.3 mA shock, and long-term memory was assessed 24 h later. H2A.Z manipulation significantly affected fear memory in females (F_4,46_ = 2.74, p = 0.04), whereby H2A.Z.1^KA^ impaired memory compared to Myc only controls (q < 0.05). No differences were found in males (Fig. 2B). Re-training mice in the same context with a single 0.5 mA shock one week later resulted in equivalent memory across all conditions, suggesting that H2A.Z.1’s effect on memory varies with strength of the learning stimulus (Supplementary Fig. 4A).

In contrast to our previous studies, H2A.Z deletion did not improve fear memory in males with 0.3 mA shock (Fig. 2B). To determine if this discrepancy is due to the weaker training compared to our previous work, a subset of males were trained with two instead of one 0.3 mA shock. With this protocol, H2A.Z deletion improved fear memory (F_4,48_ = 9.994, p < 0.001) compared to all groups (q < 0.05) except the H2A.Z.1^WT^ (p = 0.12) (Fig. 2C). In addition, the acetyl-defective H2A.Z.1^KA^ mutation impaired memory compared to all groups (all p < 0.05). To test if these effects persist with additional training, mice were trained again with a single 0.5 mA shock 1 week later. Effects of H2A.Z deletion were no longer evident, but memory remained deficient in mice expressing H2A.Z.1^KA^ (F_4,49_ = 4.347, p = 0.004) compared to all groups except H2A.Z.1^WT^ (p = 0.103) (Supplementary Fig. 4B). Together, these data show that acetyl-defective H2A.Z.1 is detrimental for fear memory, but these effects depend on the strength of the training stimulus.

#### H2A.Z deletion has unique transcriptional effects in male and female mice

H2A.Z regulates memory through changes in gene expression [11–13]. Thus, we conducted RNA sequencing on infected hippocampal tissue to identify how H2A.Z deletion and replacement impact transcription. First, we explored sex differences in Myc-tag controls and found only 137 differentially expressed genes (DEGs), most of which were more highly expressed in females than in males (Supplementary Fig. 5). To determine if males and females respond differently to H2A.Z deletion, we compared DEGs caused by H2A.Z deletion in each sex. Most upregulated genes overlapped between the sexes (3869 shared DEGs) and females had more uniquely upregulated genes (1485 unique DEGs) than males (824 unique DEGs) (Supplementary Figure 6A), suggesting that H2A.Z deletion had a larger transcriptional impact in females. Gene ontology (ShinyGo 0.77) analysis showed that genes that were upregulated by H2A.Z in both sexes were enriched for membrane, cytoplasmic ribosome, and vesicle associated terms. Upregulated DEGs unique to females were primarily enriched for membrane and structural terms (e.g., basolateral plasma membrane, extracellular matrix), whereas uniquely upregulated genes in males were enriched for nuclear terms, including nucleosome, chromosome, and protein-DNA complex (Supplementary Fig. 6A).

Genes downregulated by H2A.Z deletion followed a similar pattern, with more downregulated genes in females (1767) than in males (695 DEGs). Most genes overlapped (3071 DEGs) between the sexes and were enriched for terms relating to synaptic function, including cation channel complex, postsynaptic density, and glutamatergic synapse (Supplementary Fig. 6B). DEGs unique to females revealed primarily mitochondria related terms and a small number of synaptic terms (e.g., presynapse). In contrast, downregulated DEGs unique to males included terms related to axons, dendrites, and the synapse. Thus, H2A.Z deletion affects different functional categories for up- and down- regulated genes, with genes relevant for synaptic function being represented primarily among downregulated genes, suggesting that these genes are positively regulated by H2A.Z. Moreover, H2A.Z regulates unique gene categories in male and female mice, which may contribute to distinct effects of H2A.Z deletion on fear memory.

#### DEGs caused by H2A.Z deletion and replacement with H2A.Z.1 lysine mutants

**Fig. 3:**
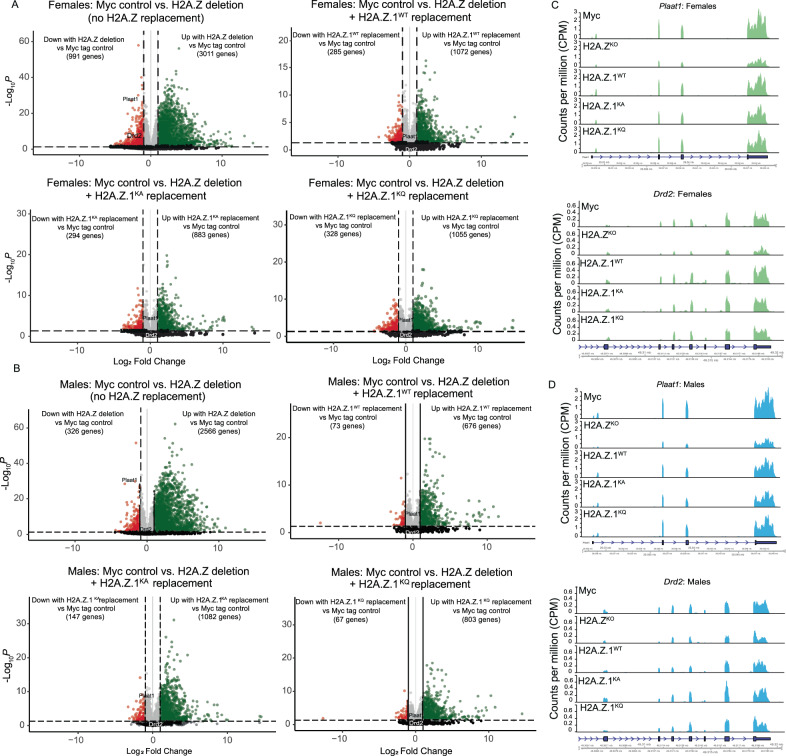
H2A.Z deletion and expression of lysine mutants impact gene expression. Volcano plots showing effects of H2A.Z deletion alone (H2A.Z^KO^) and H2A.Z deletion combined with H2A.Z.1^WT^, H2A.Z.1^KA^, or H2A.Z.1^KQ^ replacement on transcript levels in (**A**) female and (**B**) male mice. Red and green circles indicate differentially expressed genes (DEGs) with FDR < 0.05 and log_2_FC > 1 that are significantly increased or decreased relative to the Myc control group. Gray circles represent significant DEGs with FDR < 0.05 and log_2_FC < 1. Black circles indicate genes that did not meet significance criteria. Number of DEGs shown in brackets reflect FDR < 0.05 and log_2_FC > 1. Example gene tracks are shown for (**C**) female and (**D**) male mice. Data in gene tracks are expressed as Counts Per Million (CPM). N/group is as follows. Males: Myc 4, H2A.Z^KO^ 4, H2A.Z.1^WT^5, H2A.Z.1^KA^ 4, H2A.Z.1^KQ^ 5. Females: Myc 3, H2A.Z.1^KO^4, H2A.Z.1^WT^ 4, H2A.Z.1^KA^ 4, H2A.Z.1^KQ^ 3.

Next, we compared gene expression changes caused by H2A.Z deletion alone and by H2A.Z deletion combined with expression of H2A.Z.1 mutants relative to Myc-tag controls. In females, Cre-mediated co-deletion of H2A.Z.1 and H2A.Z.2 (H2A.Z^KO^) resulted in 4002 DEGs of which 24% (991 genes) were downregulated and 76% (3011 genes) were upregulated (Fig. 2A). Replacing lost H2A.Z with the wild-type form of H2A.Z.1 (H2A.Z.1^WT^) substantially restored gene expression, as evidenced by 66% fewer DEGs than H2A.Z deletion without replacement. Similarly, replacing lost H2A.Z with the acetyl-defective H2A.Z.1^KA^ or the acetyl-mimic H2A.Z.1^KQ^ also restored most dysregulated genes, resulting in 71% and 65% fewer DEGs, respectively (Fig. 3A, C; Supplementary Table 1).

H2A.Z deletion produced similar effects in males as in females, albeit with fewer total (2892) DEGs (Fig. 3A) and a smaller proportion of downregulated DEGs (11%) in males vs females (24%). Replacing lost H2A.Z with the WT form of H2A.Z.1 reduced the number of DEGs from 2892 to 749, thus restoring 74% of DEGs caused by H2A.Z deletion. Replacing lost H2A.Z with either H2A.Z.1^KA^ (57%) or H2A.Z.1^KQ^ (70%) also restored transcription (Fig. 3B, D; Supplementary Table 2), suggesting that global role of H2A.Z in transcription is similar irrespective of lysine mutation. Overall, these data show that H2A.Z has a bidirectional effect on transcription with a bias toward gene repression and that H2A.Z.1 replacement can restore effects of lost H2A.Z, irrespective of mutation. Notably, H2A.Z.1 replacement effectively restored transcriptional dysregulation caused by deleting both H2A.Z genes, consistent with greater abundance of H2A.Z.1 than H2A.Z.2 in the hippocampus (Supplementary Fig. 7).

#### Lysine mutants regulate functionally distinct transcriptional targets

To elucidate the basis for distinct effects of H2A.Z.1^KA^ and H2A.Z.1^KQ^ on memory, we identified functional categories of DEGs that were uniquely affected by each mutation. Since mutants were expressed in conjunction with H2A.Z deletion, we focused on genes that differed between H2A.Z deletion alone and H2A.Z deletion with H2A.Z.1 replacement. Specifically, we focused on genes that were dysregulated by H2A.Z deletion in one direction and by H2A.Z.1 mutant expression in the opposite direction relative to H2A.Z^KO^ mice. To facilitate gene ontology analyses, we analyzed all significant DEGs (FDR < 0.05) without applying the additional fold change (Log_2_ FC > 1) cutoff applied in Fig. 3A.

In females, 53% of genes that were downregulated by H2A.Z deletion (2582 DEGs) were not restored (i.e., upregulated compared to H2A.Z^KO^ condition) by either H2A.Z.1^KA^ or H2A.Z.1^KQ^ expression, whereas 29% were restored with both mutants. 14% were uniquely restored by H2A.Z.1^KA^ and only 4% were uniquely restored by H2A.Z.1^KQ^ expression (Supplementary Fig. 8A). DEGs that were selectively rescued by the acetyl-defective KA mutation were enriched for ontology terms relevant for synaptic function, whereas genes that were selectively restored by the acetyl-mimic KQ mutation produced a small number of ontology terms that included the cytoplasm (Supplementary Fig. 8B, C).

Of genes that were upregulated by H2A.Z deletion, 37% were not restored (i.e., downregulated) by either condition, whereas 45% were downregulated by both (Supplementary Fig. 8D). 14% were selectively restored by H2A.Z.1^KA^, including genes enriched for structural terms, and 5% were selectively restored by H2A.Z.1^KQ^, which produced only 3 ontology terms due to the low number of genes (Supplementary Fig. 8E, F).

In males, 43% of downregulated DEGs were not restored with either mutant, whereas 21% were restored by both. H2A.Z.1^KQ^ expression uniquely restored 28% of downregulated DEGs, whereas H2A.Z.1^KA^ uniquely restored only 8% of DEGs (Supplementary Fig. 9A). Despite the small number of uniquely restored genes by the acetyl-defective KA mutant, top ontology terms were related to synaptic signaling, including glutamate receptor complexes. Thus, KA is uniquely associated with expression of genes related to synaptic function in both sexes. In contrast to females, genes uniquely impacted by H2A.Z.1^KQ^ in males also included synapse-related ontology terms (Supplementary Fig. 9B, C).

Of genes that were upregulated by H2A.Z deletion, 35% were not restored by either H2A.Z.1 mutant and 43% were restored by both. H2A.Z.1^KQ^ uniquely restored 17% of DEGs and H2A.Z.1^KQ^ uniquely restored only 5% of DEGs upregulated by H2A.Z deletion (Supplementary Fig. 9D). The ontology of uniquely restored genes by H2A.Z.1^KA^ was enriched for terms related to ribosomes, whereas H2A.Z.1^KQ^ genes were enriched for immune and membrane-related terms. Together, these data show that H2A.Z.1 mutants regulate a large number of overlapping transcripts and that their unique targets may be especially important for their distinct roles in memory (Supplementary Fig. 9E, F).

#### H2A.Z.1 lysine mutants partly restore gene expression changes caused by H2A.Z deletion

**Fig. 4:**
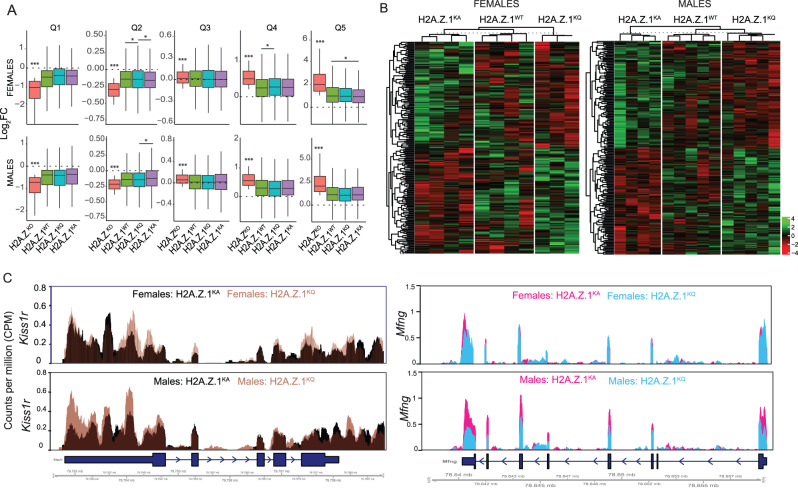
H2A.Z.1 replacement rescues gene expression changes caused by H2A.Z deletion. **A** Whisker box plots comparing DEGs from Myc controls vs. H2A.Z^KO^ sorted into quintiles by their Log_2_FC value in increasing order. ***Denotes significant difference between H2A.Z^KO^ and all other groups. *Any additional differences between individual groups are denoted with lines drawn between significantly distinct groups. P < 0.05. **B** Heat map comparing gene expression in the three H2A.Z.1 replacement groups: H2A.Z.1^KA^, H2A.Z.1^WT^ and H2A.Z.1^KQ^. Heat maps represent rlog(DESeq2) normalized raw counts that were then z-scaled. **C** Left: Gene track showing an example of H2A.Z.1^KQ^ promoting gene expression compared to H2A.Z.1^KA^ in both sexes. Right: Gene track showing an example of H2A.Z.1^KQ^ resulting in lower gene expression than H2A.Z.1^KA^ only in male mice. Data in gene tracks are expressed as Counts Per Million (CPM).

Next, we directly compared the ability of different H2A.Z.1 lysine mutations to rescue up- *versus* down-regulated DEGs by sorting H2A.Z^KO^ DEGs into quintiles ranging from most downregulated in Q1 to most upregulated genes in Q5. All H2A.Z.1 replacement conditions effectively restored down- and up-regulated genes compared to H2A.Z^KO^ in both sexes (Fig. 4A), but KA and KQ mutations differed in rescue efficiency for downregulated genes in Q2, whereby KA produced more effective rescue than KQ in males, and KQ produced a more effective rescue than KA in females (Fig. 4A). These data suggest that: (1) lysine modifications are most relevant for regulating genes that are positively associated with H2A.Z (i.e. are downregulated with H2A.Z deletion); and (2) that KA and KQ differ in rescue capacity in male and female mice.

To better understand these sex differences, we assessed the extent of differential expression caused by H2A.Z deletion in each sex. H2A.Z^KO^ produced a significant sex difference in each quintile, whereby H2A.Z deletion was less effective at downregulating and more effective at upregulating gene expression in male compared to female mice (Supplementary Fig. 10A). Assuming that genes with decreased expression are positively regulated and genes with increased expression are negatively regulated by H2A.Z under normal conditions, these data indicate that H2A.Z is a less effective transcriptional activator and a more effective transcriptional repressor in male than in female mice.

Next, we compared endogenous differences in AcH2A.Z abundance between sexes using ChIP-qPCR and found higher AcH2A.Z at a subset of genes examined, including *B2m* (t_16_ = 3.29, p = 0.002), *Th* (t_16_ = 2.05, p = 0.058), and *Fkbp5* (t_17_ = 2.36, p = 0.03), but not at *Arc, Fos*, or *Gadd45b* (Supplementary Fig. 10B). Thus, the degree of acetylation differs between sexes in a gene-specific manner and may be related to sex differences in lysine modifications on downregulated genes.

Given that replacing any form of H2A.Z rescues the robust effect of H2A.Z depletion, we reasoned that more subtle effects of lysine mutations may be masked in these conditions. Thus, we directly compared KA, KQ, and WT groups to each other to identify potential candidate genes for distinct behavioral outcomes. We identified the 100 most up- and down-regulated genes in KA relative to KQ mutations and showed that H2A.Z.1^WT^ produces an intermediate transcriptional effect between the two. Thus, distinct lysine mutations push the expression of individual genes either toward stronger or weaker expression than the WT form of H2A.Z.1, suggesting that shifts in transcription magnitude of a subset of genes may contribute to differences in behavior (Fig. 4B).

Some notable genes that emerged from this analysis (see Supplementary Tables 3, 4 for a complete list) include the kisspeptin receptor 1 (*Kiss1r*; Fig. 4C) and the voltage gated potassium channel *Kcnc1*, which are important for hippocampal plasticity and are upregulated by KQ compared to the KA mutation in both sexes. Notably, the acetyl-incompetent KA mutation also increased the expression of some genes compared to the acetyl-mimic KQ mutation, which may result in inappropriate activation of genes that suppress memory. For example, KA increased the expression of *Dkk2* (dickkopf WNT signaling pathway inhibitor 2) and *Mfng* (Beta-1,3-N-acetylglucosaminyltransferase manic fringe), which regulate Wnt and Notch signaling, respectively (Fig. 4C). Both pathways are involved in memory formation [33], suggesting that H2A.Z.1^KA^ may impair memory through inappropriate regulation of genes involved in synaptic plasticity.

#### H2A.Z and its modifications regulate alternative splicing

**Fig. 5:**
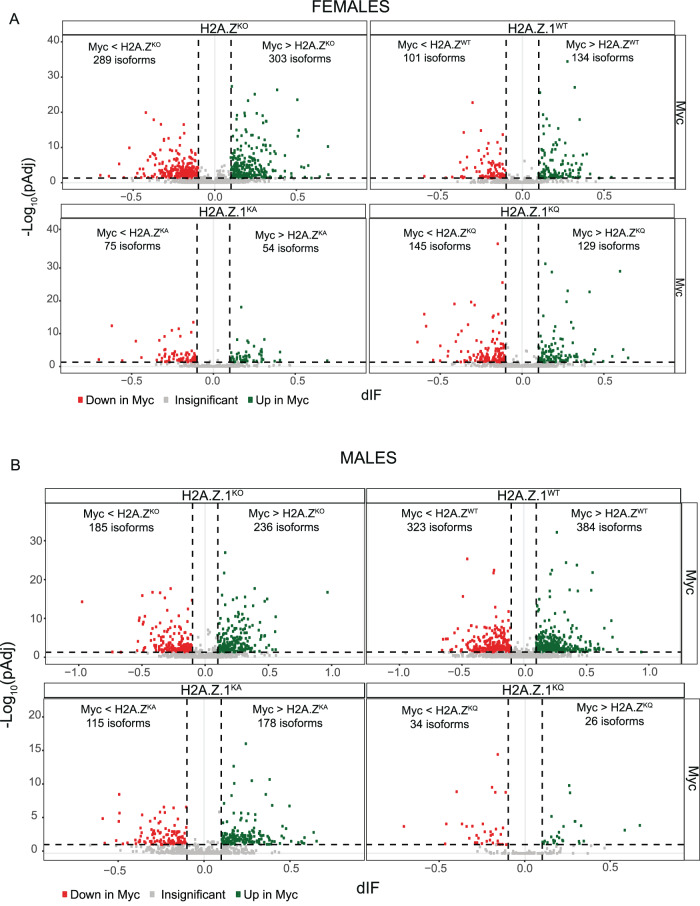
H2A.Z deletion and expression of lysine mutants impact alternative splicing. Volcano plots showing differential isoform usage for mice with H2A.Z deletion and H2A.Z.1 mutants relative to Myc controls in **A** female and **B** male mice. dIF = Differential Isoform Fraction. (FDR < 0.05).

In addition to regulating transcription, H2A.Z and PTMs of canonical histones are implicated in alternative splicing of RNA, but a role of H2A.Z acetylation in splicing is unknown [34, 35]. In females, H2A.Z deletion resulted in differential transcript usage (DTU) of 592 transcripts, and this number was reduced to 235 with H2A.Z.1^WT^, 129 with H2A.Z.1^KA^, and 274 with H2A.Z.1^KQ^ replacement. Thus, as with differentially expressed genes, DTUs caused by H2A.Z deletion were rescued by expression of any form of H2A.Z.1 (Fig. 5A). Alternative splicing impacted by H2A.Z deletion included primarily changes in Intron Retention (IR) and Alternative Transcription Termination Site (ATTS) usage, whereas replacement with H2A.Z.1^KQ^ most strongly impacted ATTS and alternative 3’ site (A3) usage (Supplementary Fig. 11A). H2A.Z.1^WT^ and H2A.Z.1^KA^ did not exhibit enrichment for any form of alternative splicing.

Next, we investigated if DTUs overlap with DEGs in females and found that some transcripts were regulated at both the expression and transcript usage levels, whereas others had either changes in expression alone, or changes in transcript usage alone. Thus, H2A.Z and its modifications can impact expression and splicing of the same or of distinct gene targets, thereby expanding the regulatory effects of H2A.Z in the brain (Supplementary Fig. 12).

In males, H2A.Z deletion resulted in 421 DTUs. In contrast to females, which exhibited a rescue in DTUs with H2A.Z.1^WT^ expression, males showed an increase to 707 DTUs compared to Myc-tag controls, whereas both H2A.Z mutants reduced the number of DTUs compared to H2A.Z deletion. Specifically, acetyl-defective H2A.Z.1^KA^ resulted in 293 DTUs, whereas the acetyl-mimic H2A.Z.1^KQ^ produced only 60 DTUs (Fig. 5B). The only form of alternative splicing that was enriched in males was alternative transcription termination site usage in both the H2A.Z.1^KA^ and H2A.Z.1^WT^ groups, whereas H2A.Z deletion and H2A.Z.1^KQ^ expression were not enriched for a particular form of splicing (Supplementary Fig. 11B). As with females, DEGs and DTUs exhibited some overlapping and some distinct gene targets, indicating that H2A.Z and its modifications can impact memory through separate effects on alternative splicing and gene expression (Supplementary Fig. 13).

## Discussion

Histone variants recently emerged as key regulators of memory that exert task- and sex-specific effects on behavior, but the basis for their variable outcomes is unknown. Here, we report that function of the histone variant H2A.Z.1 in memory is impacted by modifications of its lysine residues and related changes in transcriptional regulation, whereby mimicking H2A.Z.1 acetylation improved memory and blocking acetylation impaired memory. These effects are dependent on the strength of the training protocol, sex, and the type of learning task, suggesting that H2A.Z modulates memory strength in a lysine modification-dependent manner.

We previously showed that H2A.Z suppresses fear memory in male, but not female mice [7–9, 11–13]. Here, deletion of both H2A.Z encoding genes also had no impact on fear memory in female mice, but H2A.Z deletion in male mice only improved fear memory when shock delivery produced moderate (~40%) levels of freezing (as in our prior studies), but not when the protocol produced low (~25%) or high (80%) levels of freezing, suggesting that the primary role of H2A.Z is to modulate the strength of fear memory in the presence of a sufficient external signal. We also did not see increased freezing in male mice with history of prior shock exposure, as previously reported [9]. This did not depend on memory strength, as prior exposure to one *vs*. two 0.3 mA shocks resulted in different levels of freezing to a second 0.5 mA training session, yet neither 0.5 mA training session showed enhanced freezing with H2A.Z deletion. Thus, total levels of H2A.Z are especially important for regulating the formation of new memories and may be dispensable for altering memory strength with repeated training.

The ability of lysine mutations to regulate memory likely relates to their effects on gene expression. Our ChIP data show that AcH2A.Z colocalizes with accessible enhancers and promoters, consistent a positive effect of AcH2A.Z on transcription in various model systems [36]. However, direct mutation of lysines produced mixed effects, whereby the acetyl-incompetent KA and the acetyl-mimic KQ mutations both effectively restored up- and down-regulated DEGs. Nevertheless, direct comparisons of KA and KQ mutations revealed distinct transcriptional effects that may explain their opposing effects on memory. For example, the acetyl-defective H2A.Z.1^KA^ mutation preferentially rescued the expression of genes involved in synaptic function and glutamate signaling, suggesting that KA may impair memory by impairing synaptic plasticity.

We identified several genes that may explain differences between KA and KQ mutations in memory. Compared to the acetyl-mimic KQ, the acetyl-incompetent KA mutation reduced kisspeptin receptor 1 (*Kiss1r*) in both sexes, which binds the kisspeptin neuropeptide, a modulator of hippocampal neuronal excitability and a positive regulator of memory [37, 38]. Similarly, the KA mutation reduced expression of the voltage gated potassium channel *Kcnc1* in both sexes, suggesting that KA may also impact neuronal activity. Unexpectedly, the acetyl-incompetent KA mutation increased expression of some genes compared to the acetyl-mimic KQ, particularly in males. For example, KA increased the expression of *Dkk2*, a gene that inhibits Wnt signaling, which is critical for memory formation and synaptic plasticity [33].

The ability of KA to increase expression of some genes compared to KQ may be associated with competitive methylation and acetylation of lysines, so that the KA mutation also blocks the repressive methylation mark on H2A.Z [39]. Although we cannot assess sex differences in methylation due to lack of antibodies, sex differences in magnitude of H2A.Z acetylation suggest that male and female mice may differentially utilize distinct lysine modifications on some genes. The reason for these sex differences is unclear, but H2A.Z function is strongly intertwined with sex hormones in prostate and breast cancer cells, and its function is closely related to androgen receptor regulation in the brain [8, 40, 41]. Activation of the androgen receptor in neurons can alter H2A.Z binding [8], suggesting that interactions between H2A.Z and sex hormones may also impact sex-specific changes in the acetylation status on distinct genes.

In addition to gene expression, H2A.Z.1 and its lysine mutants also impacted alternative splicing. Although alternative splicing is associated with distinct PTMs on canonical histones [42], we are not aware of any studies linking alternative splicing with H2A.Z PTMs. However, H2A.Z occupancy can regulate alternative splicing in yeast [34, 35], and our data suggest that its role in splicing is further regulated by its lysine modifications. Alternative splicing is regulated by neuronal activity and is thought to play an important role in synaptic plasticity [43], suggesting that H2A.Z may impact behavior and neural function through impacts on both differential expression and differential splicing. Notably, differentially expressed genes and differential transcript usage only partially overlapped, indicating that H2A.Z can regulate splicing of different targets than it regulates for transcription.

### Supplementary information

Supplemental figures and methods

Table 1

Table 2

Table 3

Table 4
