# Supplementary material for: Role of the histone variant H2A.Z.1 in memory, transcription, and alternative splicing is mediated by lysine modification
Source: Neuropsychopharmacology. 2024 Feb 16;49(8):1285–95. doi: 10.1038/s41386-024-01817-2 (PMC11224360; doi:10.1038/s41386-024-01817-2)
Supplement: Supplementary file 1 — Supplemental figures and methods [file 41386_2024_1817_MOESM1_ESM.pdf]

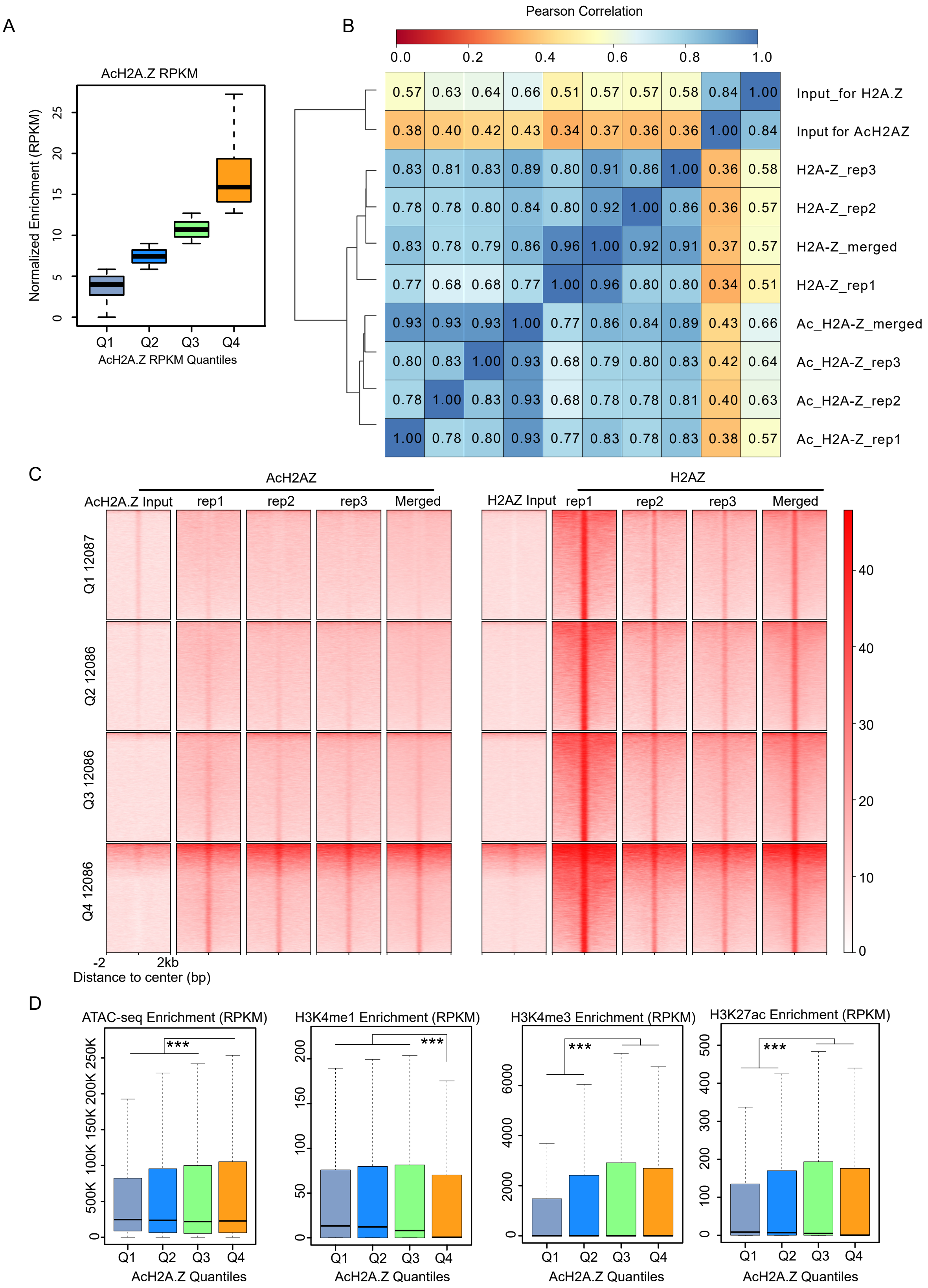

Supplemental Figure 1. Neuronal H2A.Z and AcH2A.Z ChIP-seq sample clustering and sorting into quartiles. A) Peaks for H2A.Z were separated into quartiles according to acetylated H2A.Z levels, with Q4 having the highest and Q1 having the lowest acetylation. B) Clustering of Pearson correlation measurements across all ChIP-Seq samples of AcH2A.Z and H2A.Z. C) Heatmap of RPKM enrichment for AcH2A.Z and H2A.Z at quartiles defined in Figure 1A. D) Boxplots of RKPM enrichment for chromatin accessibility, H3K4me1, H3K4me3, and H3K27ac across AcH2A.Z quartiles defined in Figure 1A.

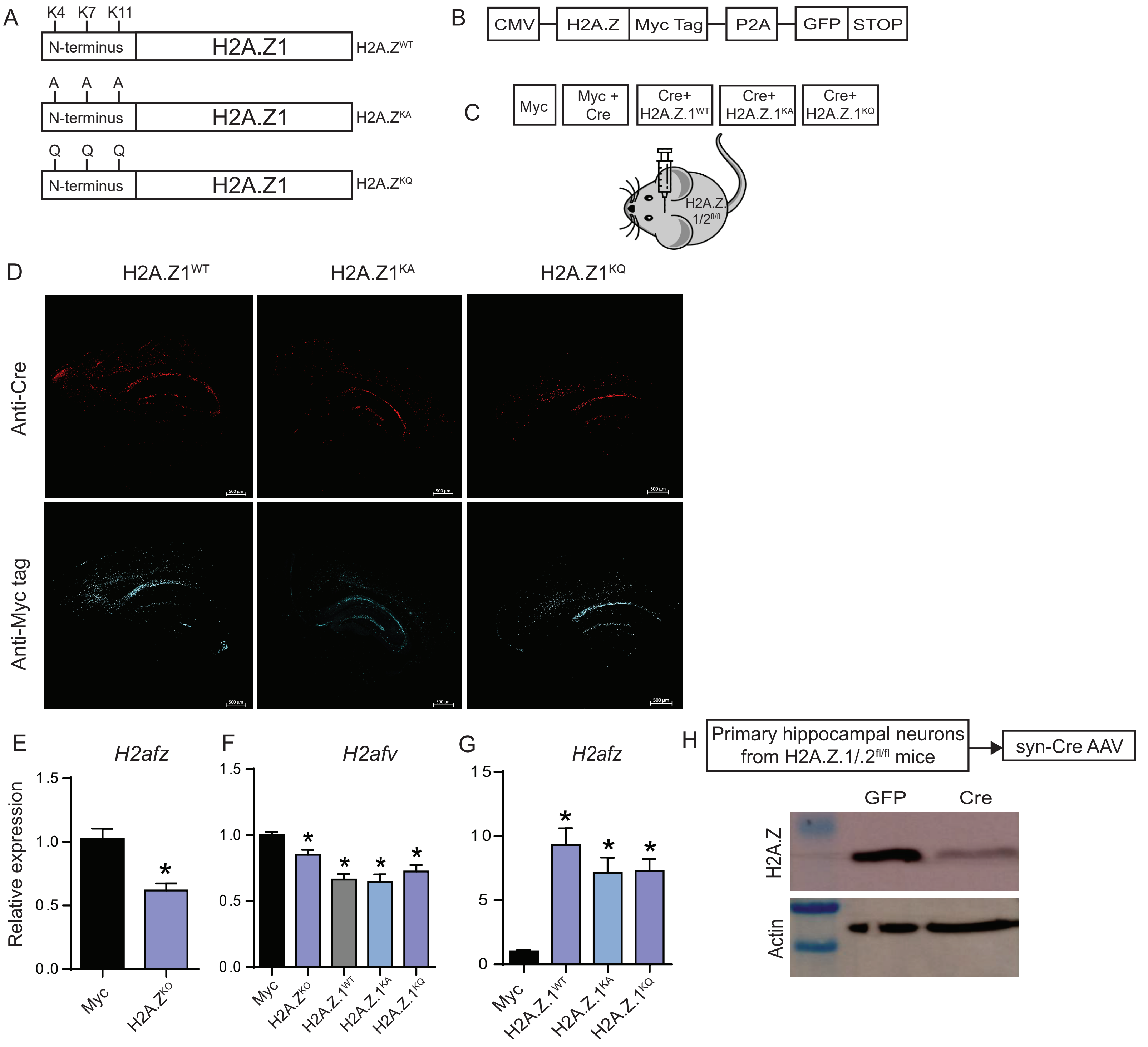

Supplemental Figure 2. *Construct design and validation*. A) H2A.Z.1 lysines 4, 7 and 11 were left intact (H2A.Z.1^{WT}), or mutated to alanine (H2A.Z.1^{KA}) to make an acetyl-defective H2A.Z.1, or mutated to glutamine (H2A.Z.1^{KQ}) to make an acetyl-mimic H2A.Z.1. B) H2A.Z.1 mutant constructs consisted of Myc tagged to H2A.Z.1 under control of the CMV promoter, followed by P2A-GFP. C) All constructs were injected into the hippocampus of mice with floxed H2A.Z.1 and H2A.Z.2 genes. Myc control group received only Myc tag construct with GFP. All other groups received a combination of Cre to delete endogenous H2A.Z and a construct to express a mutant form of H2A.Z.1. D) Cre expression was visualized using anti-Cre antibody and H2A.Z.1 construct expression was visualized using anti-Myc tag antibody. E) mRNA data confirming that *H2afz* (H2A.Z.1) expression was reduced with Cre injection in H2A.Z^{KO} mice. F) mRNA data confirming that *H2afv* (H2A.Z.2) expression was reduced with Cre injection in all groups. G) mRNA data confirming that *H2afz* expression was increased in all groups injected with mutant *H2afz* (H2A.Z.1) constructs. Data are expressed as Mean ±SEM. *$p<0.05$. H) Cre-mediated H2A.Z deletion was further validated at the protein level in cultured hippocampal neurons.

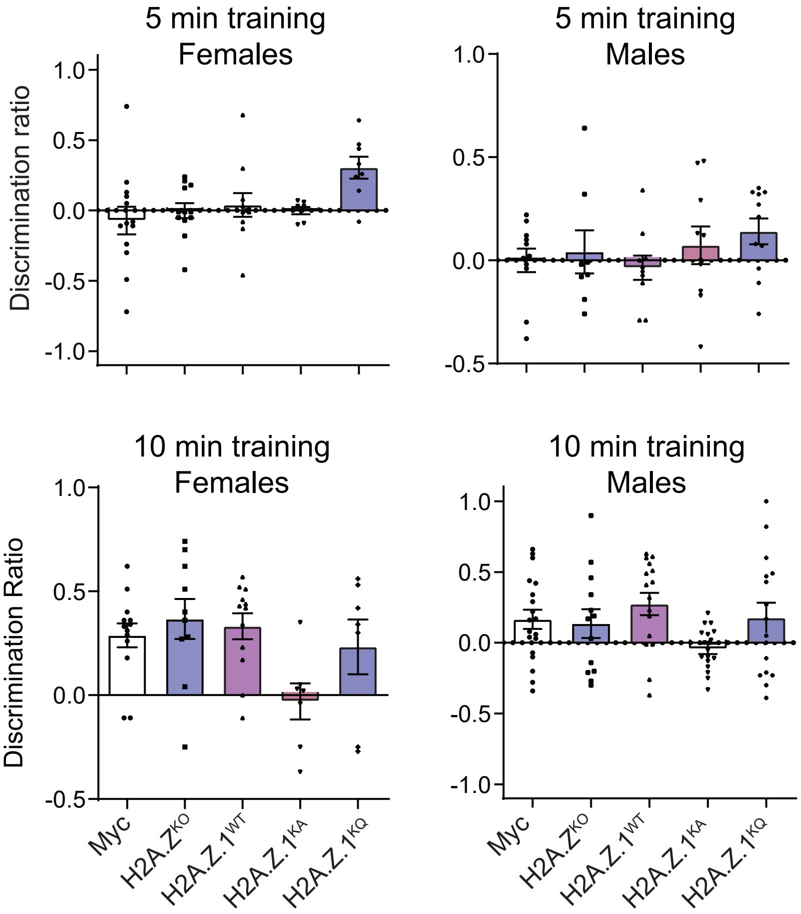

Supplemental Figure 3. OLM data split by sex. Object location memory (OLM) data were collapsed across sex in Figure 1. Here, data are shown separately for each sex. For 10 min training, there was a main effect of sex, with males having lower overall freezing rates than females.

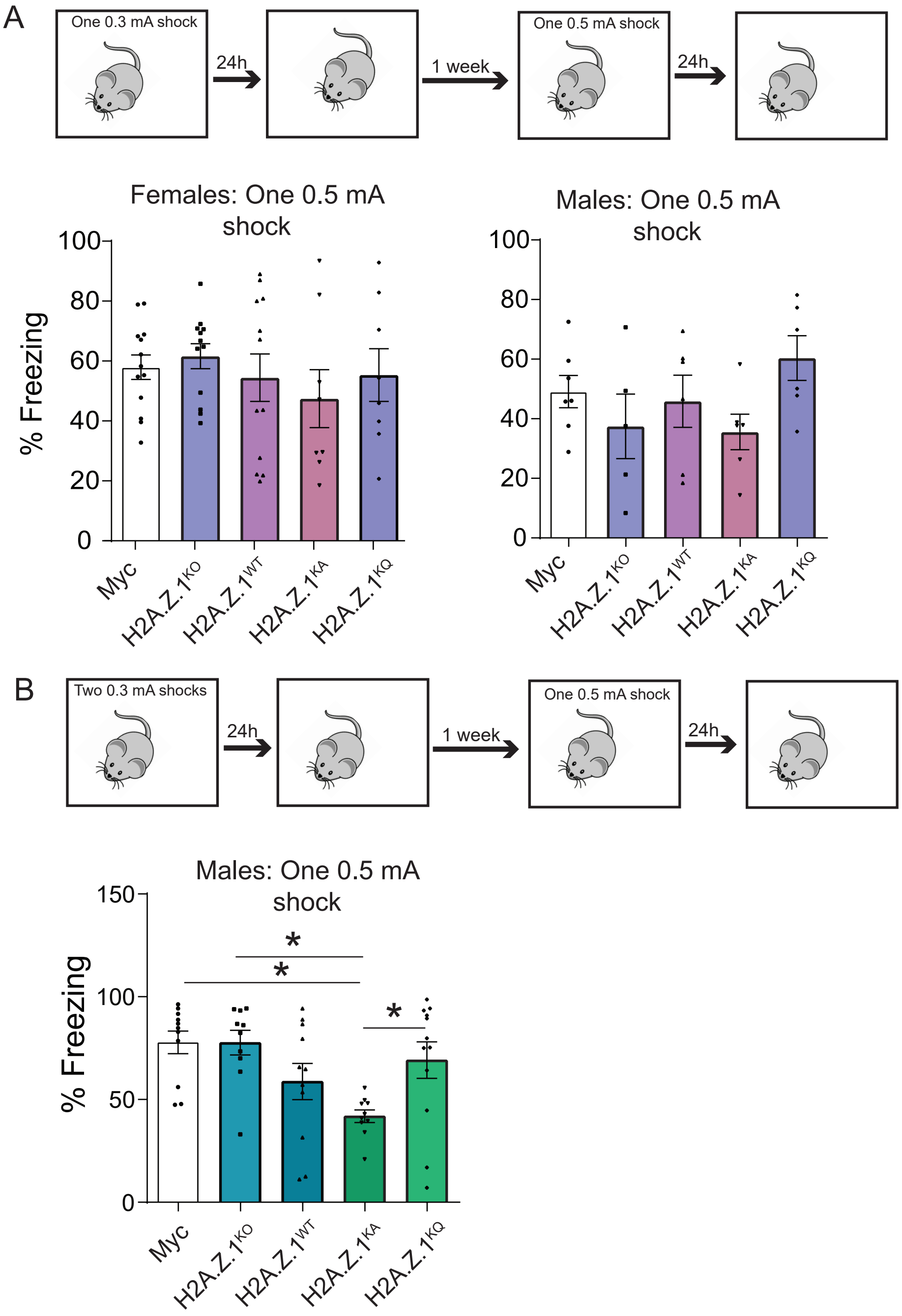

Supplemental Figure 4. Training with a second 0.5 mA foot shock blocks effects of H2A.Z mutants on memory. A) Mice trained with one 0.3 mA shock (Figure 2B) were re-trained with a single 0.5 mA shock 1 week later and memory was tested after 24 h. H2A.Z.1 mutants had no effect on memory under these conditions. B) Mice trained with two 0.3 mA shocks (Figure 2C) were re-trained with a singe 0.5 mA shock 1 week later and memory was tested after 24 h. Expression of acetyl-defective H2A.Z.1^{KA} mutant impaired memory compared to all other groups. Sample sizes are listed in Figure 2 caption. Data are expressed as Mean ±SEM. *$p<0.05$

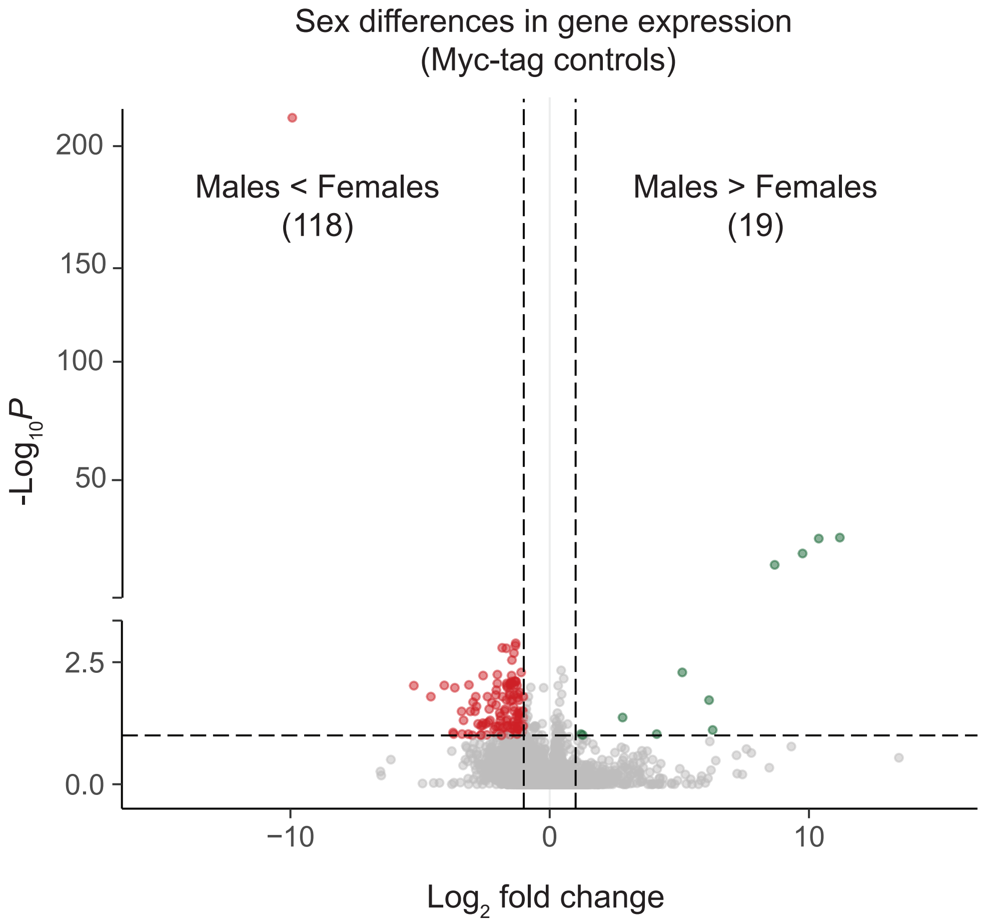

Supplemental Figure 5. Sex differences in gene expression. Gene expression was compared between male and female Myc control mice to asses differences without H2A.Z manipulation

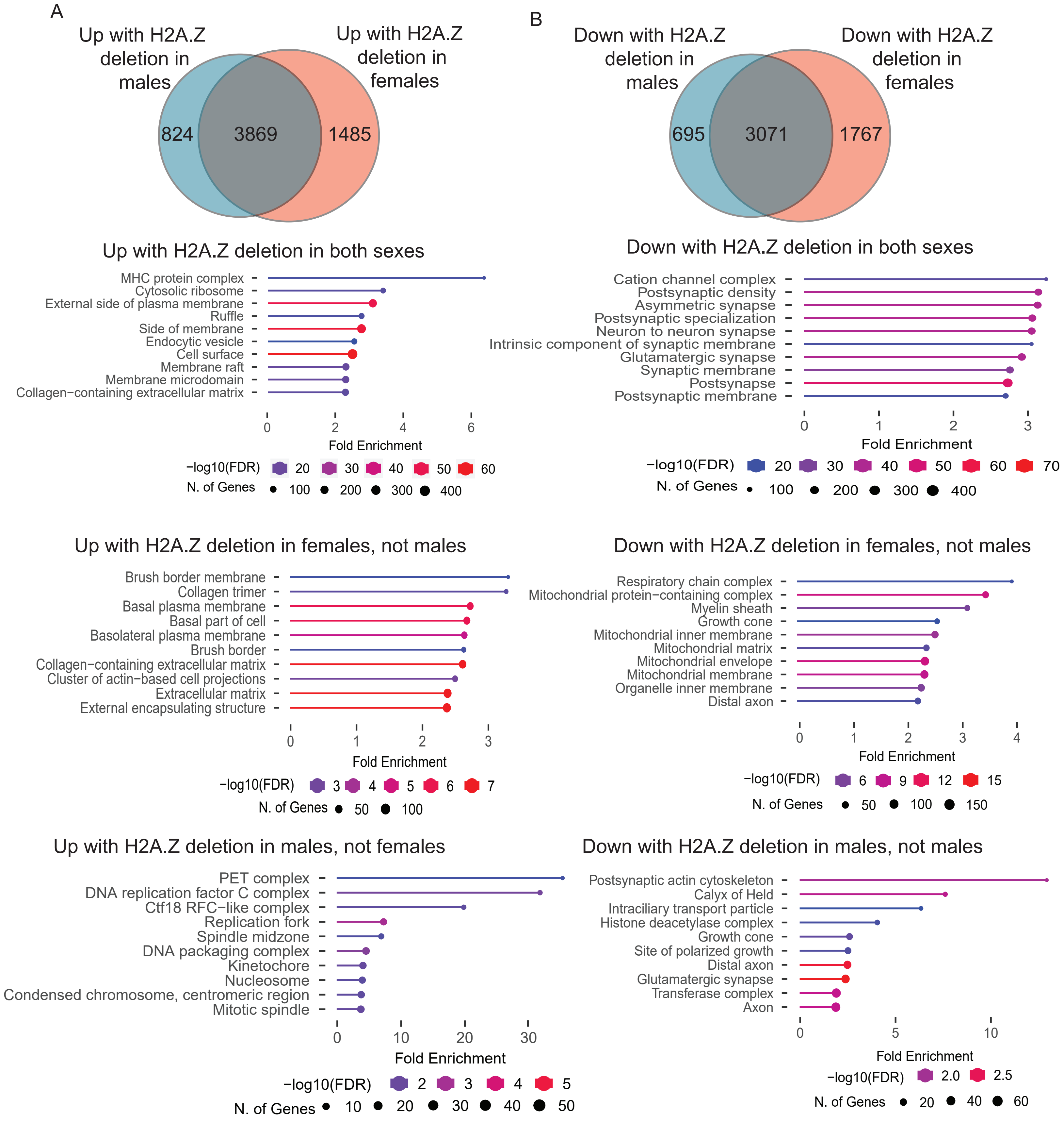

Supplemental Figure 6. Comparison of differentially expressed genes in male and female mice. A) Venn diagram showing overlap between genes upregulated by H2A.Z deletion (i.e., H2A.Z^{KO} vs. Myc) in female and male mice, followed by gene ontology for genes that are upregulated in both sexes or only in a single sex. B) Venn diagram showing overlap between genes downregulated by H2A.Z deletion (i.e., H2A.Z^{KO} vs. Myc) in female and male mice, followed by gene ontology for genes that are downregulated in both sexes or only in a single sex.

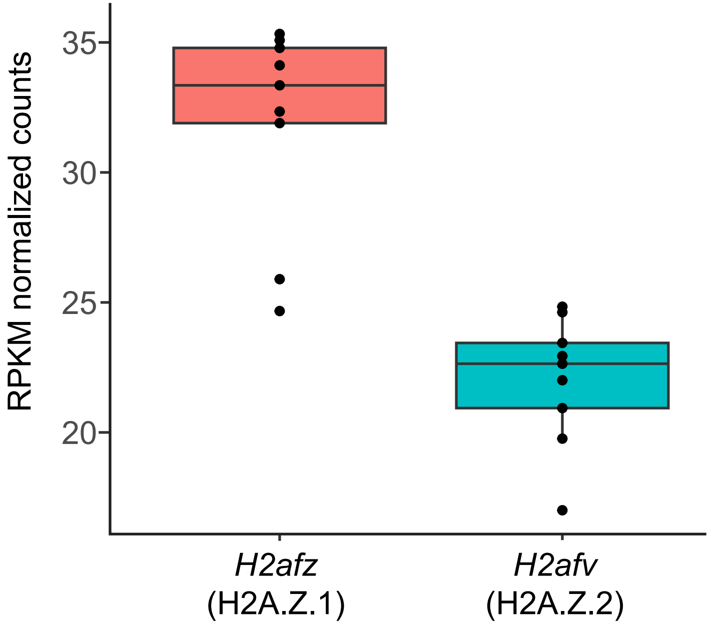

Supplemental Figure 7. ***H*2A.Z.1 is expressed more strongly than H2A.Z.2 in the mouse hippocampus*.*** Box plot comparing expression of H2A.Z.1 (*H2afz*) and H2A.Z.2 (*H2afv*) in the mouse hippocampus.

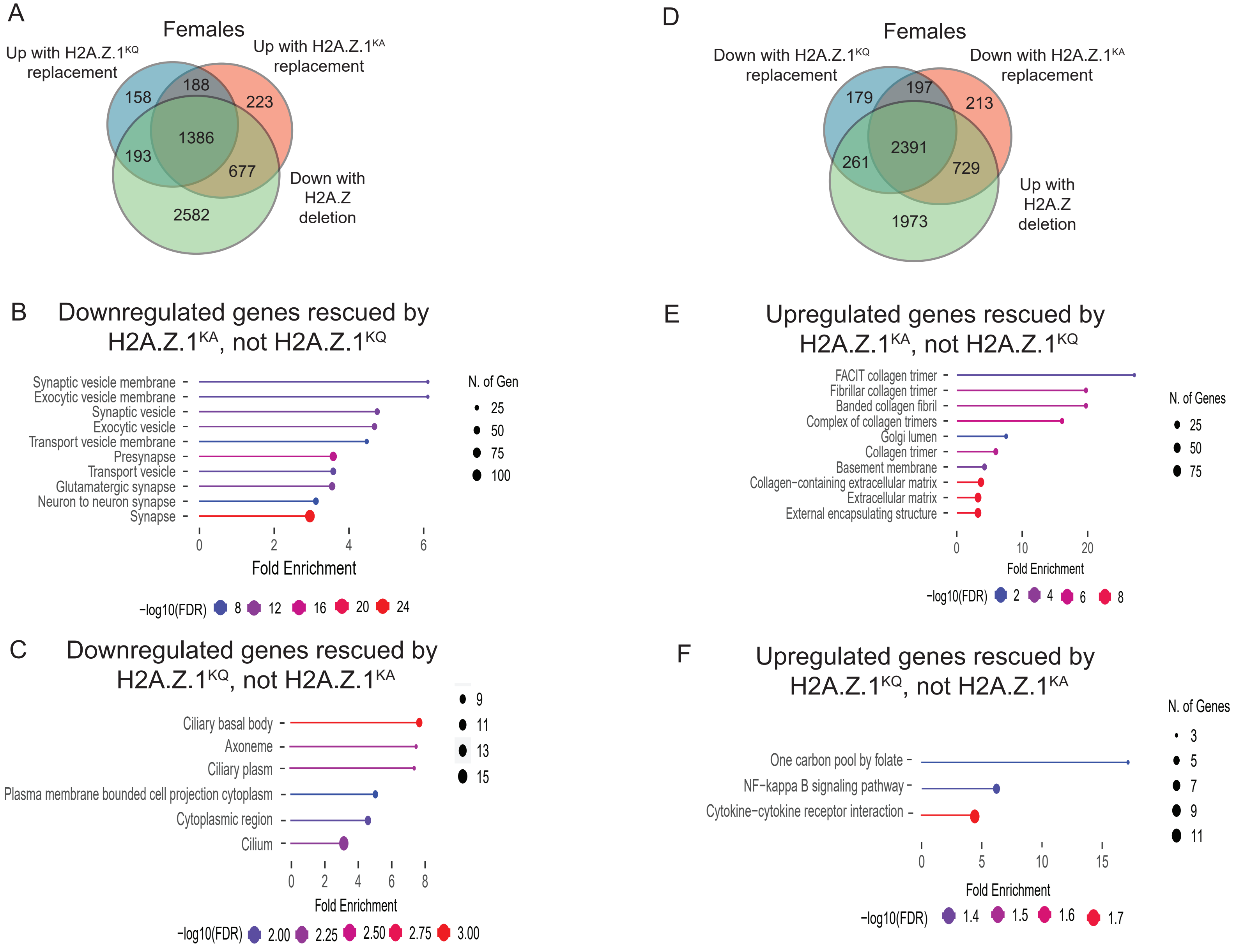

Supplemental Figure 8. Overlap in genes rescued by acetyl-mimic and acetyl-incompetent lysine mutations in female mice. A) Venn diagrams representing genes that were significantly downregulated by H2A.Z deletion and upregulated (i.e. rescued) by expression of the acetyl-mimic H2A.Z.1^{KQ} or the acetyl-defective H2A.Z.1^{KA}. To facilitate gene ontology analyses in Venn diagrams, we analyzed all significant DEGs (FDR<0.05) without applying the additional fold change (Log_2 FC>1) cutoff that was applied in Figure 3A. Thus, the number of DEGs shown reflects FDR<0.05 and no log_2FC cutoff. B) Gene ontology for genes that were selectively rescued by H2A.Z.1KA expression, but not H2A.Z.1^{KQ} expression.C) Gene ontology for genes that were selectively rescued by H2A.Z.1^{KQ}, but not by H2A.Z.1^{KA} expression.D) Venn diagrams representing genes that were significantly upregulated by H2A.Z deletion and downregulated (i.e. rescued) by expression of the acetyl-mimic H2A.Z.1^{KQ} or the acetyl-defective H2A.Z.1^{KA}. E) Gene ontology for genes that were selectively rescued by H2A.Z.1^{KA}, but not by H2A.Z.1^{KQ} expression. F) Gene ontology for genes that were selectively rescued by H2A.Z.1^{KQ}, but not by H2A.Z.1^{KA} expression.

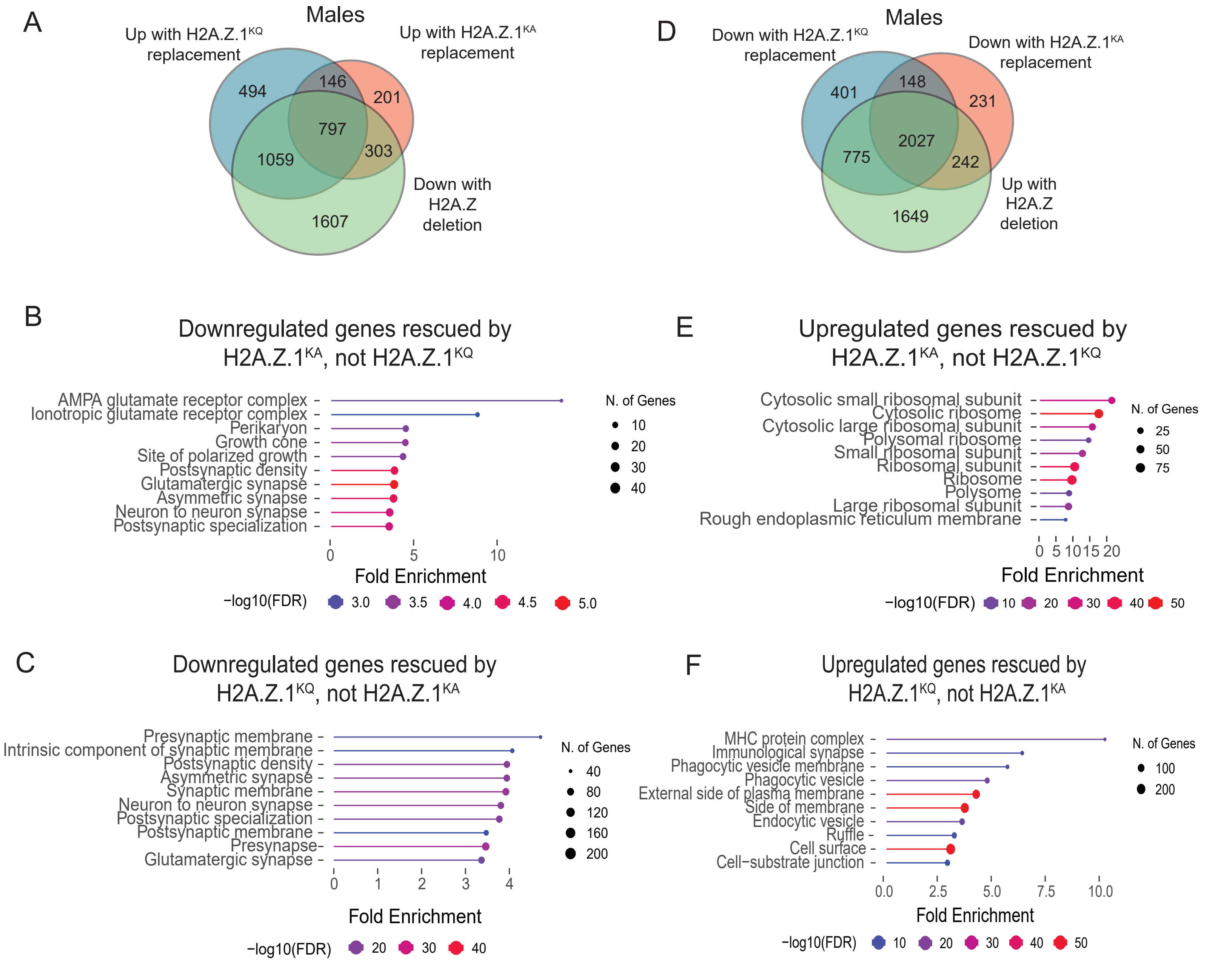

Supplemental Figure 9. Overlap in genes rescued by acetyl-mimic and acetyl-incompetent lysine mutations in male mice. A) Venn diagrams representing genes that were significantly downregulated by H2A.Z deletion and upregulated (i.e. rescued) by expression of the acetyl-mimic H2A.Z.1^{KQ} or the acetyl-defective H2A.Z.1-^{KA}. To facilitate gene ontology analyses in Venn diagrams, we analyzed all significant DEGs (FDR<0.05) without applying the additional fold change (Log$_2$ FC>1) cutoff that was applied in Figure 3A. Thus, the number of DEGs shown reflects FDR<0.05 and no log$_2$FC cutoff. B) Gene ontology for genes that were selectively rescued by H2A.Z.1^{KA} expression, but not H2A.Z.1^{KQ} expression.C) Gene ontology for genes that were selectively rescued by H2A.Z.1^{KQ}, but not by H2A.Z.1^{KA} expression.D) Venn diagrams representing genes that were significantly upregulated by H2A.Z deletion and downregulated (i.e. rescued) by expression of the acetyl-mimic H2A.Z.1^{KQ} or the acetyl-defective H2A.Z.1^{KA}. E) Gene ontology for genes that were selectively rescued by H2A.Z.1^{KA}, but not by H2A.Z.1^{KQ} expression. F) Gene ontology for genes that were selectively rescued by H2A.Z.1^{KQ}, but not by H2A.Z.1^{KA} expression.

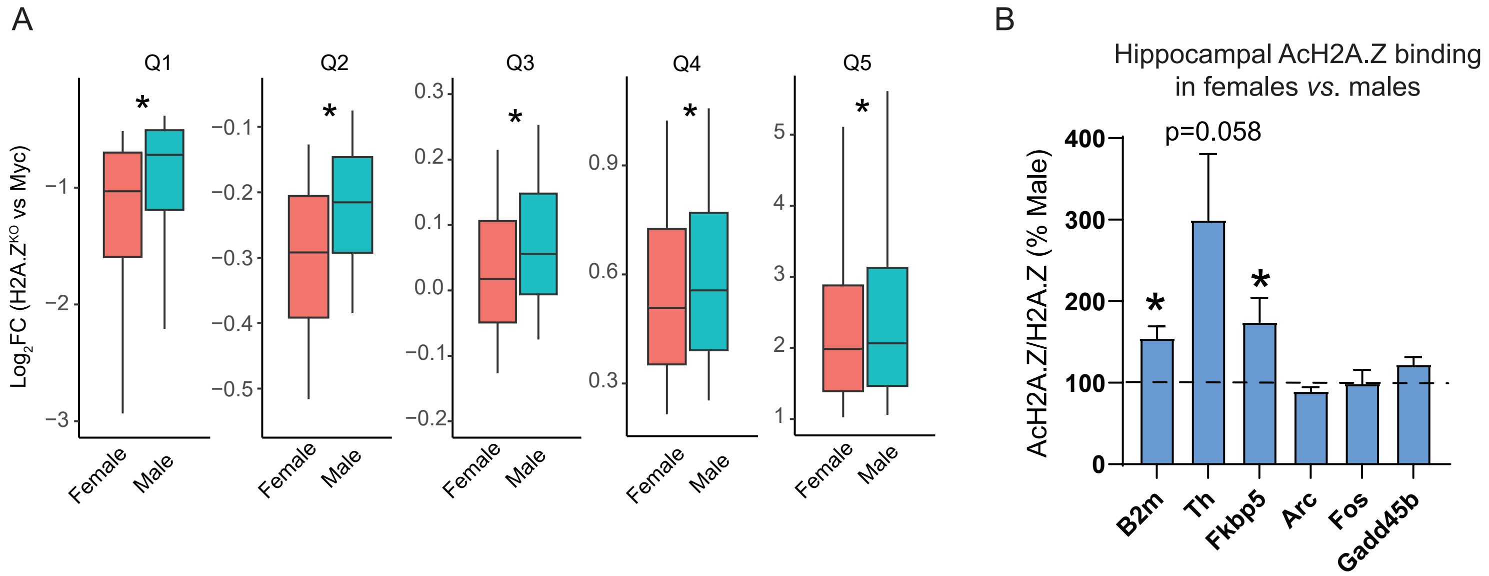

Supplemental Figure 10. Sex differences in effects of H2A.Z deletion and AcH2A.Z binding. A) Comparison of H2A.Z deletion-induced DEGs in male and female mice shows that male mice exhibit a lower magnitude of gene repression and increased magnitude of gene activation in response to H2A.Z deletion. B) AcH2A.Z ChIP signal was normalized to total H2A.Z and data are expressed as percent of signal in male mice. Blue bars represent female mice and the dashed line at 100% represents male mice. N=10/group. Data are expressed as Mean +/-SEM.

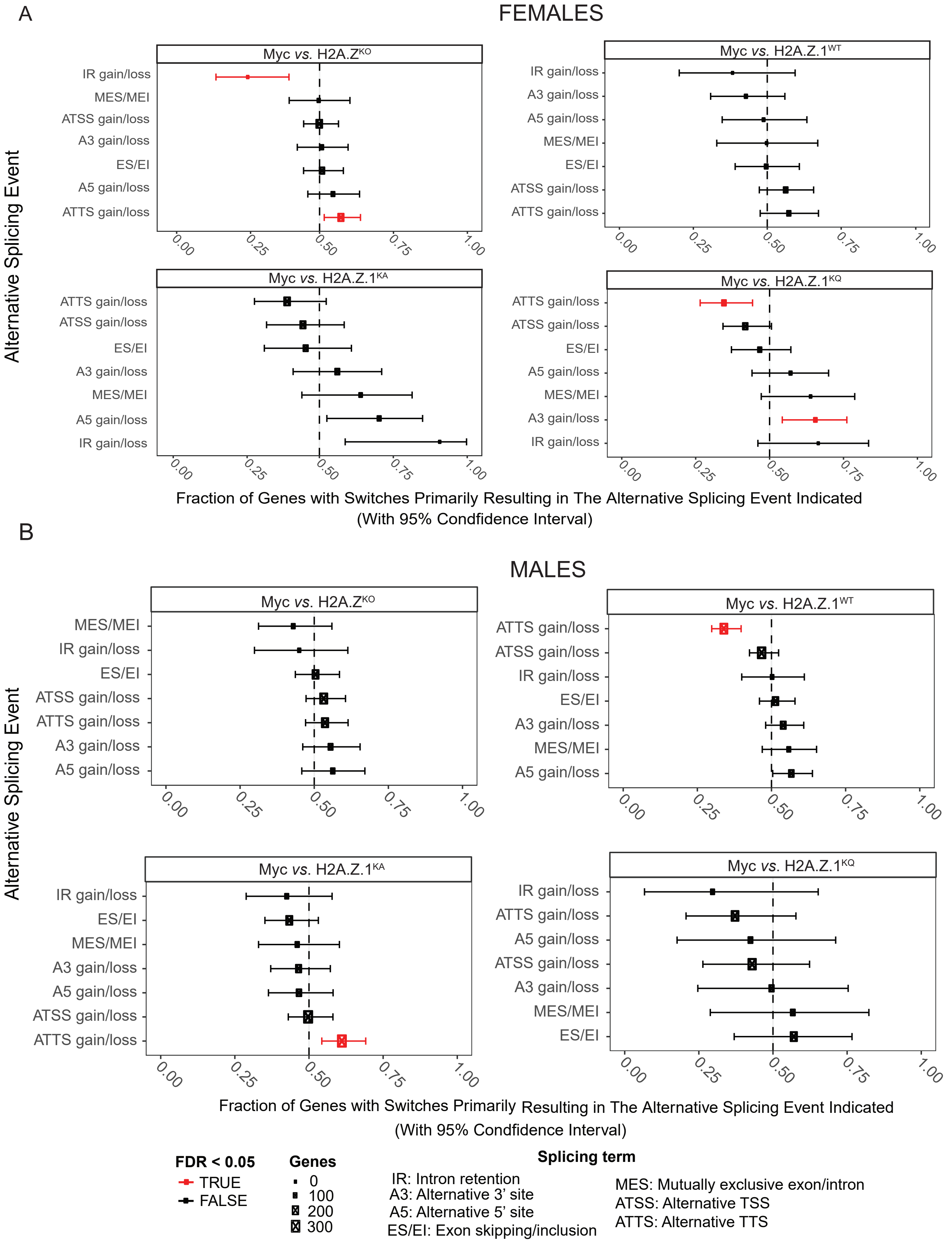

Supplemental Figure 11. Effects fo H2A.Z manipulations on the type of alternative splicing event. Plot demonstrating the type of alternative splicing change that occurred for each comparison in A) females and B) males. Red indicates a category of splicing change that was significantly enriched in the comparison of interest (FDR<0.05). Sample sizes are listed in caption for main Figure 3.

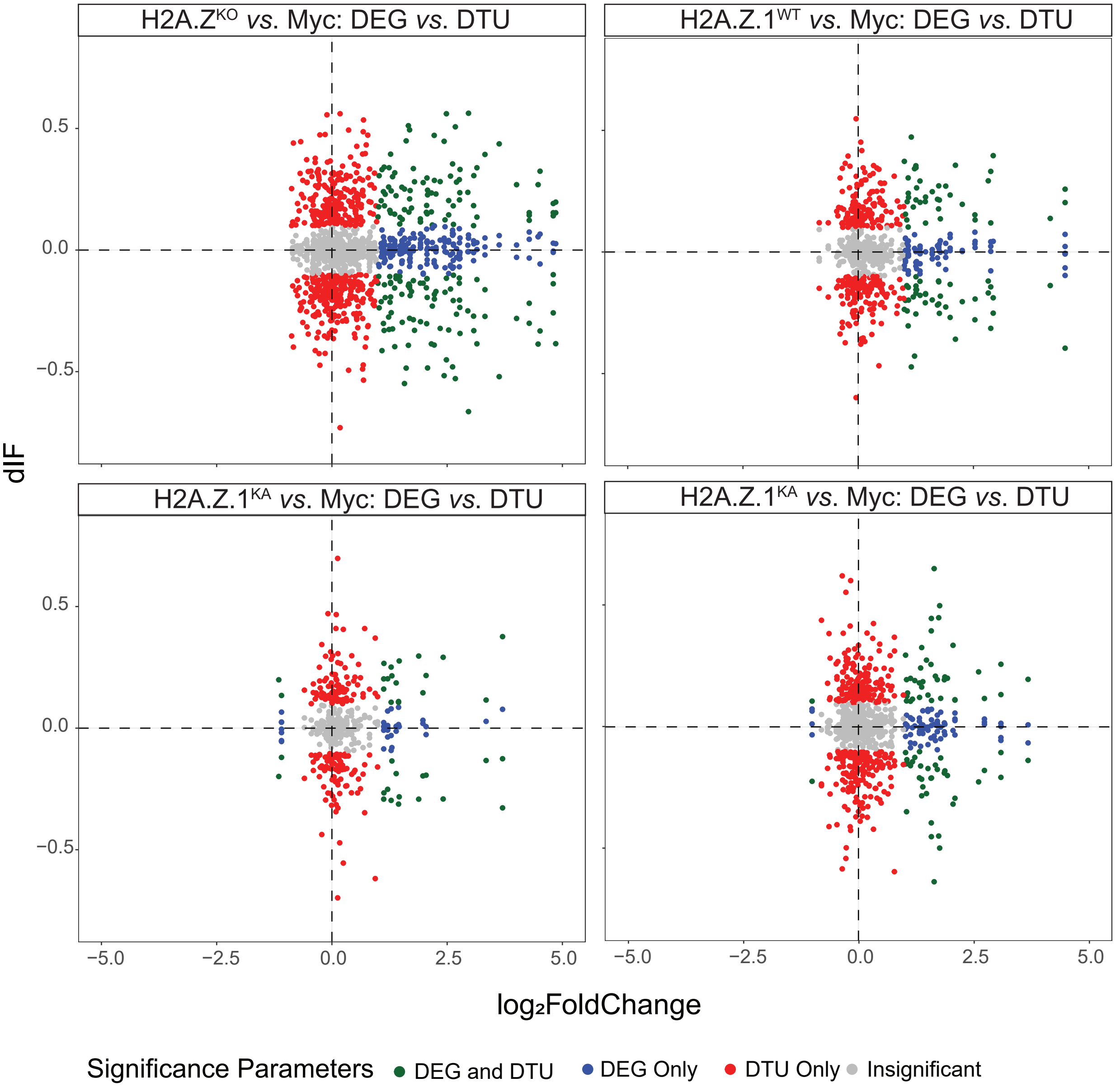

Supplemental Figure 12. ***H2A.Z deletion and mutant expression affect both overlapping and unique gene targets for differential expression and differential isoform usage in female mice.*** Plot of differentially expressed genes (DEG) and differentially transcribed units for each condition. In each treatment group, some genes were differentially spliced, but not differentially expressed (red), differentially expressed, but not differentially spliced (blue), or both differentially expressed and differentially spliced (green). dIF = differential isoform fraction.

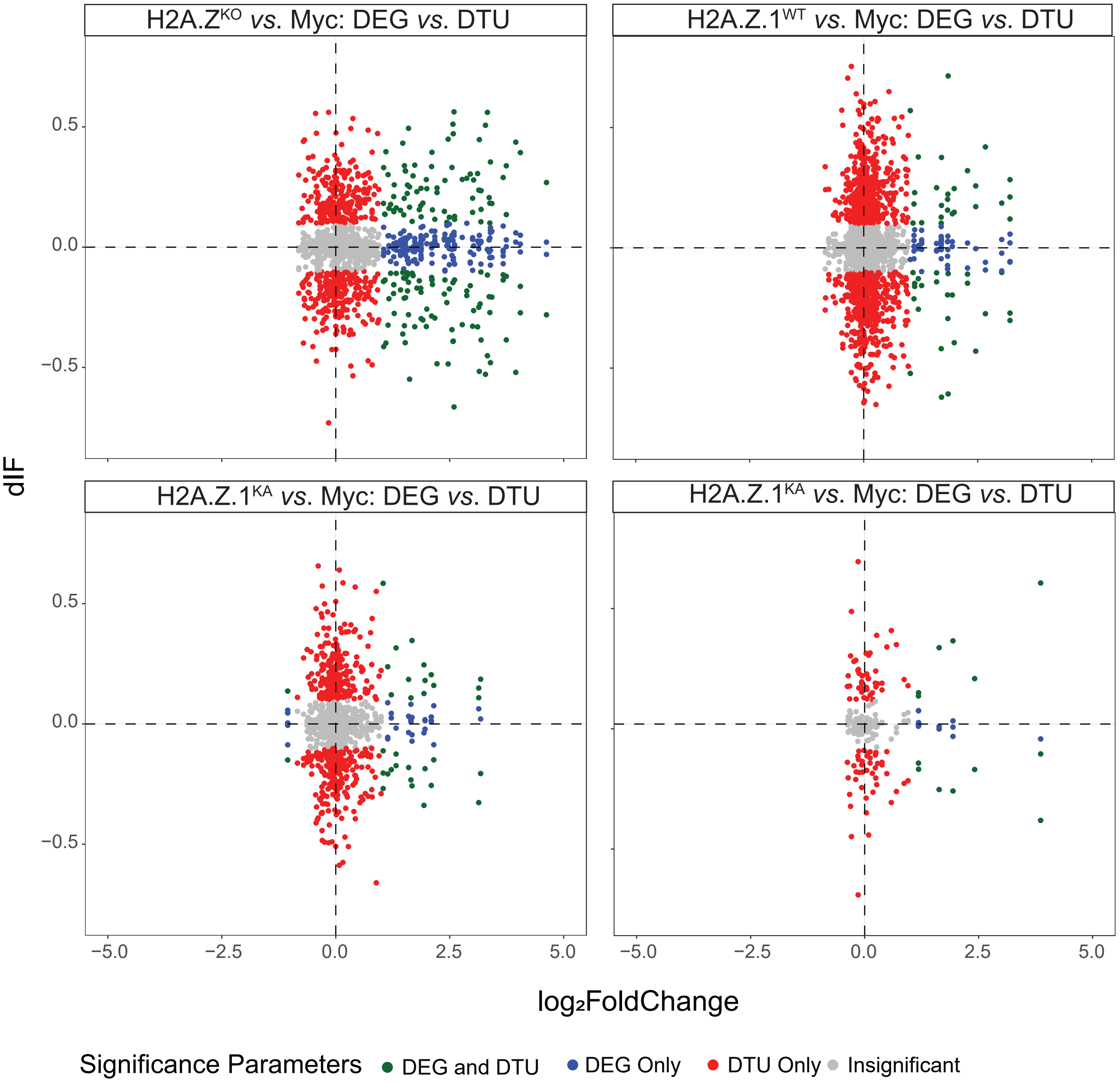

Supplemental Figure 13. H2A.Z deletion and mutant expression affect both overlapping and unique gene targets for differential expression and differential isoform usage in male mice. Plot of differentially expressed genes (DEG) and differentially transcribed units for each condition. In each treatment group, some genes were differentially spliced, but not differentially expressed (red), differentially expressed, but not differentially spliced (blue), or both differentially expressed and differentially spliced (green). dIF = differential isoform fraction.

Supplemental methods

AAV packaging

All vectors were packaged in AAV-DJ at St. Jude Children's Research Hospital. Briefly, AAV vector production was performed by PEI- (polyethyleneimine "max," Polysciences cat#24765) mediated plasmid transfection. After three days, cell pellets were harvested and lysed by repeated freeze-thawing (5x) followed by pegylation with 40% polyethylene glycol (Fisher-scientific cat# BP233-1), while supernatants were directly pegylated. Lysates and supernatants were incubated for two hours at 4°C, centrifuged at 4000g for 30 minutes at 4°C to generate a PEG containing pellet. Supernatant was discarded and pellets were resuspended in 10mM Tris, 10mM Bis-Tris-Propane. Resuspended sample was then treated with benzonase at 37°C for 1 hour and isolated via cesium chloride step gradient separation. The lower, recombinant viral genome containing fraction was collected and dialyzed three times with PBS and concentrated by 100kDA Amicon filter (Sigma Aldrich cat# UFC9100) before titering by qPCR using serial dilutions of virus compared to linearized plasmid standards [1].

ChIP sequencing

Primary hippocampal neurons were prepared and processed for AcH2A.Z ChIP sequencing as described in Stefanelli et al. 2021 [2] using anti-AcH2A.Z antibody (Millipore #ABE1363). Briefly, DIV8 hippocampal neurons were fixed o and resuspended in buffer B (50mM NaCl, 10mM Pipes pH6.8, 5mM $MgCl_2$, 1mM $CaCl_2$), incubated for 5 min at 37°C and digested using 100U of MNase (Cell Signaling, 1011S) for 15 min at 37°C. Remaining procedures are as described for ChIP qPCR above. DNA was sequenced from 3 samples per condition at Genome Quebec using HiSeq400 (~25 million reads, PE100).

Bioinformatics. Fastq files generated from AcH2A.Z ChIP-seq, and published data sets from H2A.Z ChIP-seq [2] and ATAC-seq [3] were aligned to the mm10 mouse genome using Bowtie2 (v2.2.5). Duplicates were removed using Picard (v2.5.0). Sample normalization (RPKM) was performed using Deeptools bamcoverage (v3.5.1) resulting in bigwig files that were visualized using IGV genome browser (v2.8.9). To compare AcH2A.Z ChIP-seq with published ChIP-Seq data for H3K27ac, H3K4me1, and H3K4me3, we downloaded and converted processed data [4] from mm9 to mm10 using UCSC tools liftOver (v377). Peaks were called using MACS2 bdgpeakcall (v2.2.6) (bdgpeakcall -c 25 -l 80 -g 40) and AcH2A.Z levels were scored and partitioned using multibigwigSummary (v3.5.1) with H2A.Z peaks as BED file input and normalized merged AcH2A.Z files as bigwigs. AcH2A.Z was partitioned over H2A.Z peaks in R (v4.3.2) with four quantiles defined from low to high (0-25%, 25%-50%, 50%-75%, 75%-100%). Homer annotatePeaks (v4.11) was used to assess genomic context of all AcH2A.Z quantiles using default settings. Nearest genes identified within each quartile were inputted into ClusterProfiler (v4.10.0) for gene ontology. Boxplots and bubble charts were made using standard tools in R and ggplot2 (v3.4.3). Deeptools plotHeatmap (v3.5.1) was used to generate heatmaps for all normalized H2A.Z and AcH2A.Z bigwig replicates. Deeptools plotProfile was used to generate average aggerate plots from published H3K4me3, H3K27ac, H3K4me1 and ATAC-seq across each quantile. Deeptools multiBigwigSummary and plotCorrelation (v3.5.1) were used to generate Pearson correlation heatmap at promoter regions. Affinity Designer was used to adjust labels, fonts, and text sizes. Bedtools random (v2.30.0) was used to generate random regions with 1kb in length. Bedtools fisher (v2.30.0) was used for calculating the odds ratio and two-tailed hypergeometry testing, to determine the significance of overlap between promoters or intergenic CpG Islands with each AcH2A.Z quartile. Promoters were defined as regions spanning 1Kb around

gene TSS. Bed files for CpG islands were downloaded from the UCSC genome browser and parsed to select regions that do not intersect with promoters.

References

1. Brimble, M.A., et al., *Preventing packaging of translatable P5-associated DNA contaminants in recombinant AAV vector preps.* Mol Ther Methods Clin Dev, 2022. **24**: p. 280-291.
2. Stefanelli, G., et al., *The histone chaperone Anp32e regulates memory formation, transcription, and dendritic morphology by regulating steady-state H2A.Z binding in neurons.* Cell Rep, 2021. **36**(7): p. 109551.
3. Fernandez-Albert, J., et al., *Immediate and deferred epigenomic signatures of in vivo neuronal activation in mouse hippocampus.* Nat Neurosci, 2019. **22**(10): p. 1718-1730.
4. Gjoneska, E., et al., *Conserved epigenomic signals in mice and humans reveal immune basis of Alzheimer's disease.* Nature, 2015. **518**(7539): p. 365-9.
